# Sign of APOBEC editing, purifying selection, frameshift, and in-frame nonsense mutations in the microevolution of lumpy skin disease virus

**DOI:** 10.3389/fmicb.2023.1214414

**Published:** 2023-11-14

**Authors:** Perumal Arumugam Desingu, T. P. Rubeni, K. Nagarajan, Nagalingam R. Sundaresan

**Affiliations:** ^1^Department of Microbiology and Cell Biology, Indian Institute of Science, Bengaluru, India; ^2^Department of Veterinary Pathology, Madras Veterinary College, Chennai, India; ^3^Veterinary and Animal Sciences University (TANUVAS), Chennai, India

**Keywords:** lumpy skin disease virus, frameshift mutations, in-frame nonsense mutations, selection pressure, purifying selection, APOBEC mutations

## Abstract

The lumpy skin disease virus (LSDV), which mostly affects ruminants and causes huge-economic loss, was endemic in Africa, caused outbreaks in the Middle East, and was recently detected in Russia, Serbia, Greece, Bulgaria, Kazakhstan, China, Taiwan, Vietnam, Thailand, and India. However, the role of evolutionary drivers such as codon selection, negative/purifying selection, APOBEC editing, and genetic variations such as frameshift and in-frame nonsense mutations in the LSDVs, which cause outbreaks in cattle in various countries, are still largely unknown. In the present study, a frameshift mutation in LSDV035, LSDV019, LSDV134, and LSDV144 genes and in-frame non-sense mutations in LSDV026, LSDV086, LSDV087, LSDV114, LSDV130, LSDV131, LSDV145, LSDV154, LSDV155, LSDV057, and LSDV081 genes were revealed among different clusters. Based on the available complete genome sequences, the prototype wild-type cluster-1.2.1 virus has been found in other than Africa only in India, the wild-type cluster-1.2.2 virus found in Africa were spread outside Africa, and the recombinant viruses spreading only in Asia and Russia. Although LSD viruses circulating in different countries form a specific cluster, the viruses detected in each specific country are distinguished by frameshift and in-frame nonsense mutations. Furthermore, the present study has brought to light that the selection pressure for codons usage bias is mostly exerted by purifying selection, and this process is possibly caused by APOBEC editing. Overall, the present study sheds light on microevolutions in LSDV, expected to help in future studies towards disturbed ORFs, epidemiological diagnostics, attenuation/vaccine reverts, and predicting the evolutionary direction of LSDVs.

## Introduction

Lumpy skin disease is a double-stranded DNA virus infection caused by the Lumpy skin disease virus (LSDV), which is causing significant economic losses in many countries by causing decreased milk production, abortions, infertility, reduced sperm quality, and damaged hides in cattle (Abutarbush et al., 2015; Lu et al., 2021; Kumar and Tripathi, 2022; Shumilova et al., 2022). Further, LSDV, encoding approximately 156 proteins with a genomic size of ~151,000 bp, belongs to the genus Capripoxvirus in the family Poxviridae and is closely related genomically and antigenically to Goatpox virus (GTPV) and Sheeppox virus (SPPV) (Sprygin et al., 2020a; Ma et al., 2022). LSDV is a transboundary disease that mainly affects cattle and buffaloes and also affects springbok, impala, and giraffe (Young et al., 1970; Le Goff et al., 2009; Namazi and Khodakaram Tafti, 2021); it causes <10% mortality and 0%–90% morbidity in cattle (Abutarbush et al., 2015; Lu et al., 2021; Kumar and Tripathi, 2022; Shumilova et al., 2022), causing huge production loss and livelihood of people/country that depend on the farming industry, and farm, which affects the food industry and consumers who depend on the industry, has been recognized as a notifiable disease by the World Organisation for Animal Health (OIE) (Lu et al., 2021; Ma et al., 2022; Shumilova et al., 2022; Wang et al., 2022).

Interestingly, LSDV was endemic in Africa and caused outbreaks in Middle Eastern countries (Kumar, 2011; Chibssa et al., 2021; Krotova et al., 2022a,b; Ma et al., 2022); and in recent years, it was detected in 11 European countries, majorly Greece, Turkey, Serbia, and Russia (Abutarbush et al., 2015; Tasioudi et al., 2016; Sevik and Dogan, 2017; Sprygin et al., 2018a,b; Ma et al., 2022), as well as Asian countries including China, Taiwan, Hong-Kong, India, Thailand, Bangladesh, and Nepal (Abutarbush et al., 2015; Acharya and Subedi, 2020; Badhy et al., 2021; Lu et al., 2021; Tran et al., 2021; Ma et al., 2022). The vaccine-like recombinant strains with genetic recombination signatures of Neethling- and KSGP-based LSDV vaccines were detected in Kazakhstan and bordering areas of Russia and China (Sprygin et al., 2018a,b, 2020b; Vandenbussche et al., 2022), followed by recombinant viruses detected in Taiwan, Hong-Kong, and Thailand (Tran et al., 2021; Flannery et al., 2022; Ma et al., 2022), whereas in India, wild-type viruses closely related to Kenyan isolate caused the outbreak in 2019–2022 (Badhy et al., 2021; Bhatt et al., 2023; Kumar et al., 2023). It is also noteworthy that vaccine-associated viruses with 67 single nucleotide polymorphisms (SNPs) caused outbreaks in South Africa in the 1990s (van Schalkwyk et al., 2020). Generally, LSD viruses are attenuated by serial passages in the unnatural host or unnatural host cells such as chicken eggs, rabbit kidney cells, and lamb kidney cells and are produced as vaccine strains (Wallace and Viljoen, 2005; Tuppurainen et al., 2021).

Generally, virus evolution takes place through host adaptation, host codon selection (Desingu and Nagarajan, 2022; Desingu et al., 2022a,b), positive selection, negative/purifying selection pressures, and APOBEC enzymes (Bonvin et al., 2006; Bulliard et al., 2011; Warren et al., 2015; Gigante et al., 2022; Isidro et al., 2022; Pecori et al., 2022); importantly, host APOBEC enzyme editing plays an essential role in the restriction of retroviruses (HIV), DNA viruses such as monkeypox virus (Mpoxv), hepatitis B virus (HBV) and human papillomavirus (HPV) (Bonvin et al., 2006; Bulliard et al., 2011; Warren et al., 2015; Gigante et al., 2022; Isidro et al., 2022; Pecori et al., 2022). Also, in the evolution of poxviruses, gene inactivation is generated through frameshift mutations and in-frame nonsense mutations.; Especially 2,502 accessory genes of Orthopoxviruses are inactivated, 795 genes are inactivated through frameshift mutations, 947 genes are through within gene deletions, and 759 genes are inactivated through complete deletion (Senkevich et al., 2021). Furthermore, gene loss/inactivation (frameshift mutations or genomic region deletion or in-frame nonsense mutations) in the poxviruses is expected to determine the host range, evasion of the host immunity, pathogenicity, and virulence (Biswas et al., 2020; Senkevich et al., 2020, 2021; Brennan et al., 2022). In a few viruses recently detected in the LSDV outbreak, LSDV019 and LSDV144 genes have been reported to be two fragments, LSDV019a, LSDV019b, and LSDV144a, LSDV144b, respectively, through frameshift mutations (Kumar et al., 2023). In this situation, frameshift mutations, in-frame nonsense mutations, codon selection pressure, positive selection pressure, negative/purifying selection pressure, and APOBEC editing among different clusters in LSD viruses are largely unknown. By understanding such evolutionary development, it will be helpful to determine which types of viruses and their genetic variation are spreading in different geographical areas.

In this present study, frameshift mutations and in-frame nonsense mutations in LSD viruses were found by systematically analyzing almost all complete genome sequences of LSD virus in the NCBI public database, and it has brought to light that there is a sign of selection pressure, purifying selection, and APOBEC editing in the microevolution of these viruses.

## Methods

### Data collection and data curation

In the present study, almost all complete genome sequences of the LSD viruses were retrieved from the NCBI public database. The complete genome sequences were aligned using the MAFFT 7.407_1 alignment program with the parameters of Gap extend penalty-0.123 and Gap opening penalty-1.53 (Katoh and Standley, 2013; Mareuil et al., 2017; Lemoine et al., 2019). Further, the quality of the alignments was checked and curated using the LAST Plot hits utilizing the MAFFT version 7 of the online server tool with a score of 39 (*E* = 8.4e^−11^)^1^ (Katoh and Frith, 2012; Katoh et al., 2019). When these nucleotide sequences were aligned, it was revealed that the three sequences OK422492.1/India/2019/Ranchi-1/P10, OK422493.1//India/2019/Ranchi-1/P3, and ON400507.1/208/PVNRTVU/202 submitted to the NCBI public database from India had the highest nucleotide diversity. Subsequently, LAST hits plot analysis revealed that these three sequences were submitted as reverse complement compared to NCBI reference sequences NC_003027.1_LSDV_NI-249 (Supplementary Figures S1A–D). Therefore, throughout this present study, we have converted these three sequences into reverse complements and subjected them to analysis. Also, we have retrieved LSDV complete genome sequences from SRA run files SRR21590382, SRR21590384, SRR21590385, SRR21590386, and SRR21590383 related to LSDV submitted to NCBI public database from India and subjected to analysis. Briefly, these SRA run files were subjected to quality control using Trimmomatic (Bolger et al., 2014) to filter and remove low-quality reads and potential adopters sequences from the reads (Vilsker et al., 2019). From these quality control passed reads, the virus-specific reads were filtered using the protein-based alignment method DIAMOND (Buchfink et al., 2015), and *de novo* assembled using metaSPAdes (Bankevich et al., 2012), *de novo* assembled virus sequences recognized using Blastx and Blastn in the NCBI RefSeq virus database. Further, individual contig was aligned in the advanced genome aligner (AGA) (Deforche, 2017), and consensus variant caller GATK/BcfTools were used in the analyses. Confirm annotated variants and SNPs and mismatches with Raw reads were presented in Supplementary Data 10.

### Phylogenetic analysis

In the present study, the complete genome nucleotide sequences of LSD viruses were aligned in MAFFT 7.407_1, and then phylogenetic analysis was performed in PhyML 3.3_1 (Galaxy Version 3.3_1) (Mareuil et al., 2017). The GTR (evolutionary model), discrete gamma model (categories with the *n* = 4), empirical (equilibrium frequencies), subtree pruning and regraphing with tree topology search with tree topology, model parameter, and branch length, and then branch support were tested with approximate Bayes branch. Subsequently, the generated phylogenetic tree with the above parameters was visualized in the interactive tree of life (iTOL)-v5 (Letunic and Bork, 2021).

### Net between group mean distance analysis

In the present study, the whole genome nucleotide sequences of the LSD viruses were aligned in MAFFT 7.407_1, and then the aligned nucleotide sequences were used for the NBGMD analysis in MEGA7 (Kumar et al., 2016) using the Kimura two-parameter model with the transitions + transversions substitution, gamma distribution (shape parameter = 5), gaps/missing data were deleted by pairwise deletion, and finally, the standard errors for the NBGMD analysis were calculated by the bootstrap of 1,000 replicates. The calculated standard error in the analysis was presented above the diagonal of the result table.

### SimPlot analysis

The SimPlot 3.5.1 (Desingu et al., 2022a,b) tool was used to determine the per cent identity/similarities among the different clusters of LSD viruses against reference sequences. In this study, the whole genome nucleotide sequences of the LSD viruses were aligned in MAFFT 7.407_1. Then the aligned nucleotide sequences were exported to SimPlot 3.5.1 tool for the subsequent analysis using the Kimura (two-parameter) method and base pairs of the window of 500 at a base-pair step of 50.

### Recombination detection program analysis

The complete genome sequences of LSDVs were aligned in the MAFFT 7.407_1 and then exported to RDP4 (Martin et al., 2015) for the recombination analysis. The recombination analysis was performed using default parameter values for the RDP, GENECONV, BOOTSCAN, Chimaera, 3seq, SISCAN, and MaxChi methods, and a minimum of four approaches was assessed for possible recombination using a Bonferroni corrected *p*-value cut-off (0.05).

### Measurement of nucleotide/amino acid mismatch, transition/transversion, and silent/non-silent mutation

The nucleotide/amino acid mismatch, transition/transversion, and silent/non-silent mutations were measured among the different clusters of LSD viruses against the reference sequence at the complete genome levels, gene levels, and genes that are transcribed in the forward and reverse directions by the Highlighter tool (Keele et al., 2008) with or without similarity sorting of the sequences, with/without treating the gaps as a character, and the reference sequences used in the analysis were displayed in the respective figures. Additionally, the nucleotide/amino acid mismatch was also visualized by the online Variant Visualizer,^2^ and the reference sequences used in the analysis were displayed in the respective figures.

### Measurement of APOBEC motif mutations and d*N*/d*S* ratio

The APOBEC motif mutations in the LSD viruses’ complete genome sequences and genes transcribed in the forward/reverse directions were determined in the Hypermut 2.0 tool with the customized options (Rose and Korber, 2000) against the reference sequences, and the reference sequences used were displayed in the respective figures. Next, the d*N*/d*S* ratio in the LSD virus’s genes transcribed in the forward/reverse directions was measured in the SNAP v2.1.1 (Ota and Nei, 1994; Ganeshan et al., 1997; Korber, 2000). The reference sequences used in the analysis were displayed in the respective figures.

### Nucleotide sequence composition analysis

The nucleotide composition (A%, T%, G%, and C%) of the LSD viruses’ complete genome sequences and genes transcribed in the forward/reverse directions were determined in MEGA7 (Kumar et al., 2016) and Automated Codon Usage Analysis (ACUA) Software (Vetrivel et al., 2007).

### Effective number of codons

The effective number of codon usage from 61 codons for the 20 amino acids for the LSD viruses’ genes transcribed in the forward/reverse directions was determined in version 6 of the DNA Sequence Polymorphism (DnaSP) software (DnaSP 6) (Rozas et al., 2017).

### ENc-GC3s plot

In ENc-GC3s plot analysis, the ENc values are plotted against the third position of GC3s of codon values for the LSD viruses’ genes transcribed in the forward/reverse directions. The expected curve was determined as recommended in previous publications (Wang et al., 2018; Tian et al., 2020). The ENc values and the GC3s of codon values were measured in version 6 of the DNA Sequence Polymorphism (DnaSP) software (DnaSP 6) (Rozas et al., 2017).

### Parity rule 2-bias plot

For parity rule 2 (PR2)-bias, the AT bias [A3/(A3 + T3)] is plotted against GC-bias [G3/(G3 + C3)] in the LSD viruses’ genes transcribed in the forward/reverse directions. The A3, T3, G3, and C3 values of nucleotide sequences were obtained by using ACUA Software (Vetrivel et al., 2007).

## Results

### Genetic diversity at the complete genome levels

**Figure 1:**
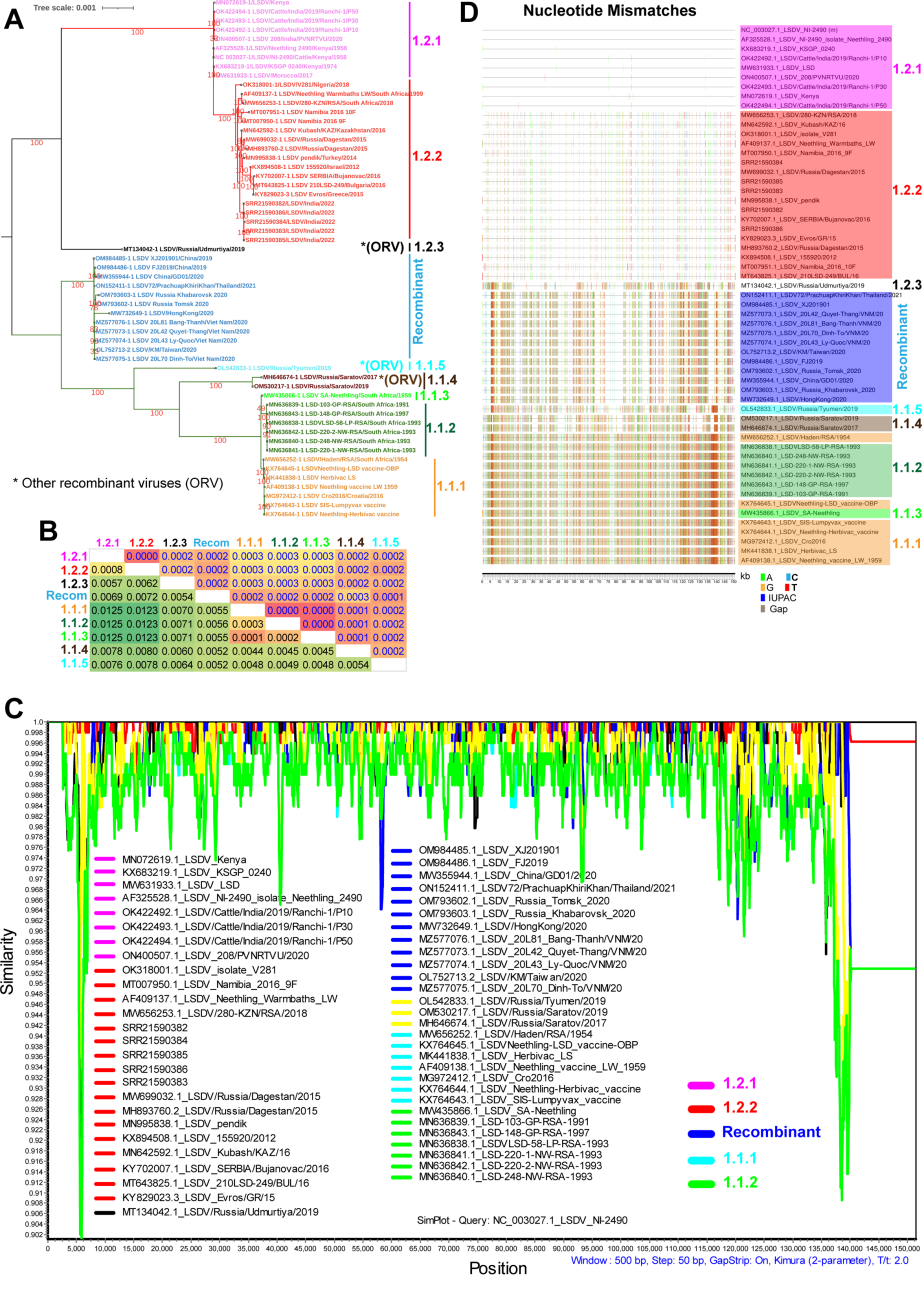
Complete genome nucleotide sequence level genetic diversity in LSD viruses. **(A)** Whole genome nucleotide sequences based on phylogenetic analysis of LSDV sequences separated them into different clusters. **(B)** The whole genome nucleotide sequences based NBGMD analysis revealed less than 1.25% nucleotide diversity among clusters of the LSD virus. The details of the virus’s sequence in different clusters are presented in **(A)**. The estimated standard error was displayed above the diagonal in the table. **(C)** The SimPlot analysis depicts the multiple regions of recombination with mostly lesser than 0.25% genetic diversity among clusters of the LSD virus at the whole genome nucleotide sequence levels. The NC_003027.1 sequence is used as a query sequence in this analysis. **(D)** Nucleotide mismatches at the level of whole genome nucleotide sequences have been depicted in different clusters of LSD viruses. The NC_003027.1 sequence was used as a reference in this analysis.

To understand the phylogenetic relationship of LSD viruses at the whole genome level, we retrieved almost all LSDV complete genome sequences from the NCBI public database and performed phylogenetic analysis. In the phylogenetic analysis, viruses related to wild-type were clustered into cluster-1.2, viruses related to vaccine into cluster-1.1, and recombinant viruses clustered into recombinant-cluster (Figure 1A), as reported by the previous studies (van Schalkwyk et al., 2020; Krotova et al., 2022a,b; Ma et al., 2022). Consistent with this, we also observed the recombinant events in the recombinant viruses by recombination detection program (RDP) analysis (Supplementary Figures S1–S3). Further, with the topology of the phylogenetic tree, LSD viruses, we have divided cluster-1.1 viruses into five sub-clusters, namely cluster-1.1.1 to 1.1.5 (Figure 1A), and similarly cluster-1.2 viruses into three sub-clusters namely cluster-1.2.1 to 1.2.3 (Figure 1A) (Ma et al., 2022; van Schalkwyk et al., 2022; Bhatt et al., 2023). Among these, sub-cluster-1.1.1 and 1.1.3 contain vaccine viruses, sub-cluster-1.1.2 contains vaccine-associated virulence field strains detected in South Africa, and sub-clusters-1.1.4 and 1.1.5 contain vaccine-associated recombinant virulence field strains detected in Russia (Figure 1A). Similarly, cluster-1.2.3 of the Udmurtiya strain is also a recombinant virus (Figure 1A). Furthermore, the recombinant viruses in clusters-1.1.4, 1.1.5, and 1.2.3 are different from the recombinant viruses belonging to the recombinant cluster that were detected in Russia, China, Thailand, Hong Kong, Taiwan, and Vietnam in 2019–2021 (Figure 1A). For better readability, hereafter recombinant cluster viruses are recognized as recombinant, and other recombinant viruses such as Utmurtia, Saratov, and Tyumen are recognized as 1.2.3, 1.1.4, and 1.1.5, respectively. In particular, wild-type viruses in sub-cluster-1.2.1 have been detected only in African countries and India. Finally, wild-type viruses in sub-cluster-1.2.2 are detected in African countries, Russia, Kazakhstan, Turkey, Israel, Greece, Bulgaria, and India (Figure 1A), however, Greece and Bulgaria have eradicated LSDV through mass vaccination.

Furthermore, Net Between Group Mean Distance (NBGMD) analysis revealed less than 1.25% nucleotide diversity among wild-type viruses (sub-clusters-1.2.1 and 1.2.2) and vaccine and vaccine-derived viruses (sub-clusters-1.1.1, 1.1.2, and 1.1.3) (Figure 1B). Viruses in the recombinant cluster exhibited 0.52%–0.72% nucleotide diversity with viruses in other clusters, including wild-type and vaccine strains (Figure 1B). Since there is only a very low level of nucleotide diversity between different clusters, it is clear that microevolution has occurred between these clusters. After this, we conducted a similarity plot analysis to determine which genomic regions have the highest genetic diversity among these clusters. This analysis revealed less than 0.25% genetic diversity in almost all genomic regions between distinct clusters, and viruses in the recombinant cluster exhibited recombination with wild-type and vaccine viruses in multiple genomic regions (Figure 1C) (Krotova et al., 2022a,b; Ma et al., 2022). Also, similar to similarity plot analysis, nucleotide mismatches analysis revealed that viruses in the recombinant cluster alternately exhibited nucleotide similarity with wild-type and vaccine viruses in multiple genomic regions and that nucleotide differences were primarily due to SNPs (Figure 1D) and majorly, transition mutations (Supplementary Figure S4). Since viruses in the recombinant cluster alternately express SNPs with wild-type and vaccine viruses in multiple genomic regions, LSDV-vaccine strains are attenuated (likely due to disruption of virulence genes to attenuate the virus) mainly by the passage in the unnatural host or unnatural host’s cells (Wallace and Viljoen, 2005; Tuppurainen et al., 2021), and this virus infects cattle, buffaloes, springbok, impala and giraffe (Young et al., 1970; Le Goff et al., 2009; Namazi and Khodakaram Tafti, 2021). A recent study using a short-read next-generation sequencing method explains the possibility of the origin of recombinant LSDVs through the homologous recombination of the Neethling-like LSDV vaccine strain and KSGP-like LSDV vaccine strains in the vaccine (Vandenbussche et al., 2022).

### Mutations altering the open reading frames

**Figure 2:**
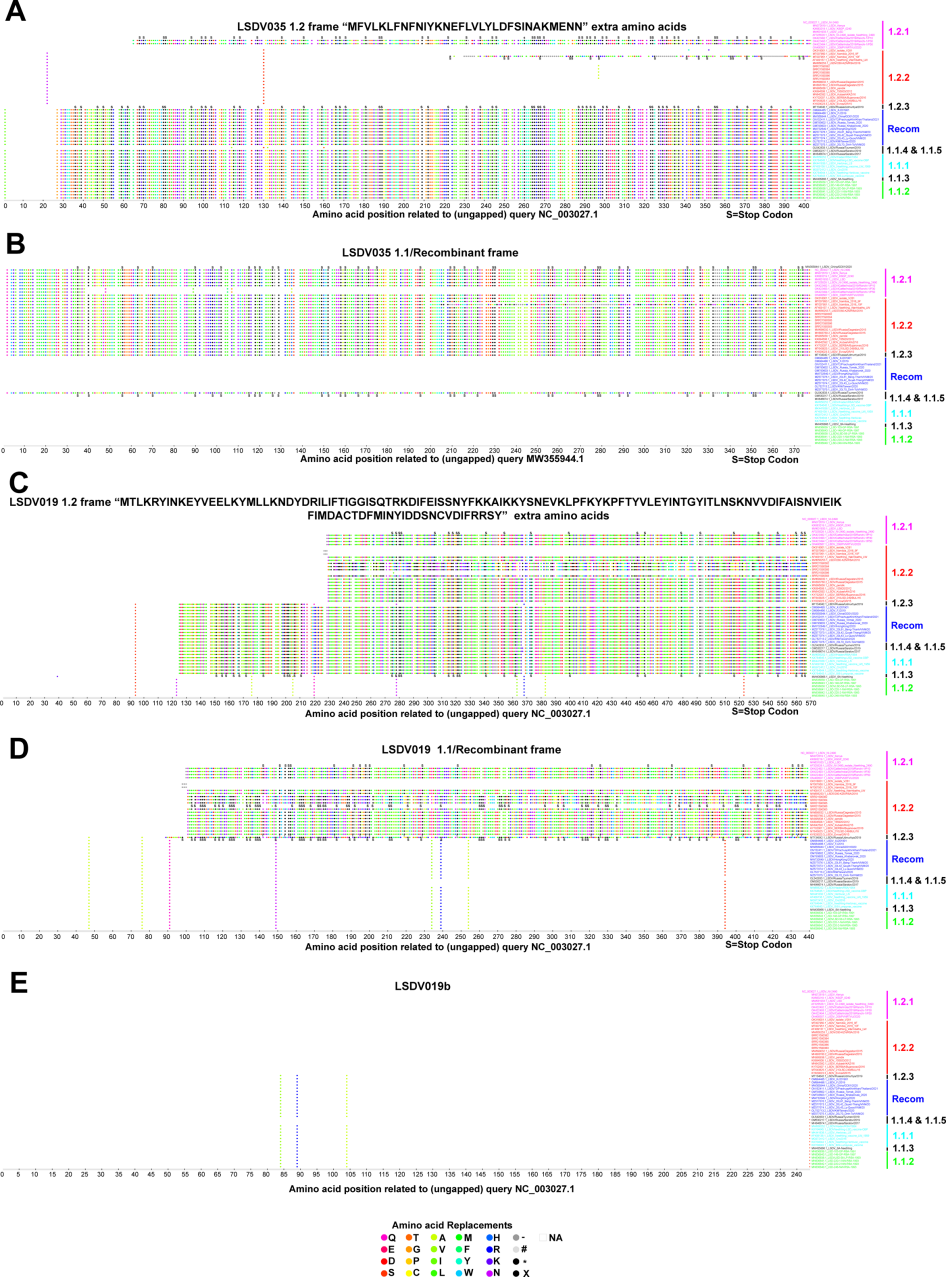
Frameshift mutations in LSDV035 and LSDV019 genes. **(A,B)** Frameshift mutations in the LSDV035 gene **(A)** cluster-1.2 frame, and **(B)** cluster-1.1/recombinant frame. **(C–E)** Frameshift mutations in the LSDV019 gene **(C)** cluster-1.2 frame, **(D)** cluster-1.1/recombinant frame, and **(E)** LSDV019b frame. Details of sequences used in this analysis are provided in Supplementary Data 1.

**Figure 3:**
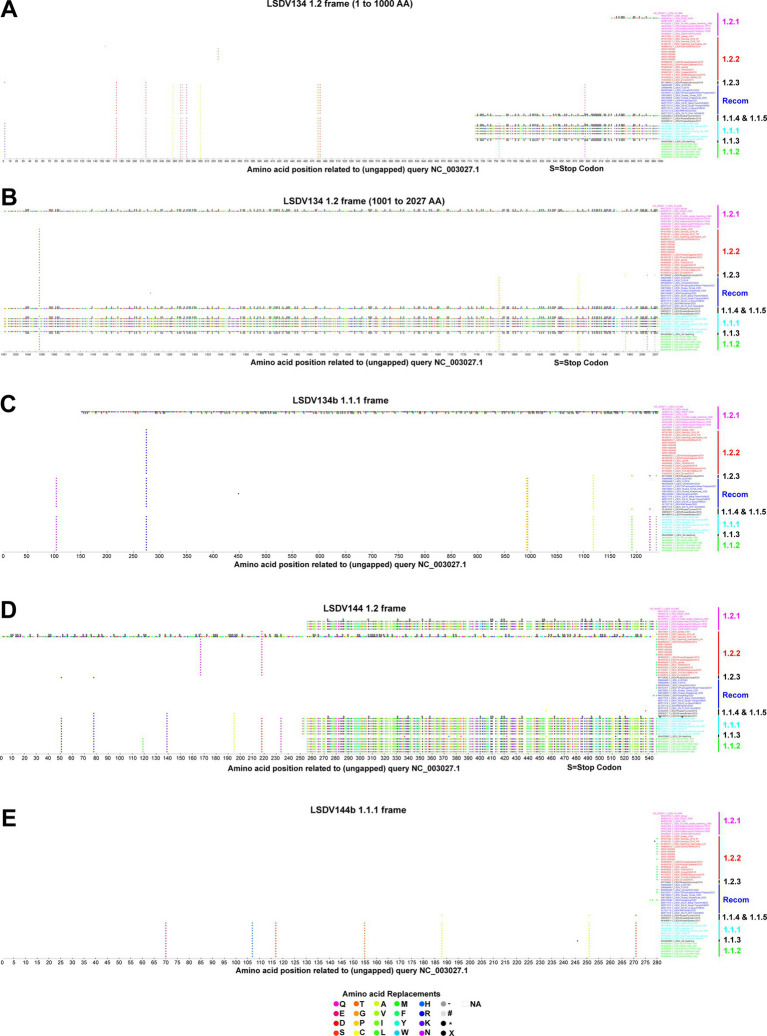
Frameshift mutations in LSDV134 and LSDV144 genes. **(A–C)** Frameshift mutations in the LSDV134 gene **(A,B)** cluster-1.2 frame, and **(C)** cluster-1.1.1 frame. **(D,E)** Frameshift mutations in the LSDV144 gene **(D)** cluster-1.2 frame, and **(E)** cluster-1.1.1 frame. Details of sequences used in this analysis are provided in Supplementary Data 1.

**Figure 4:**
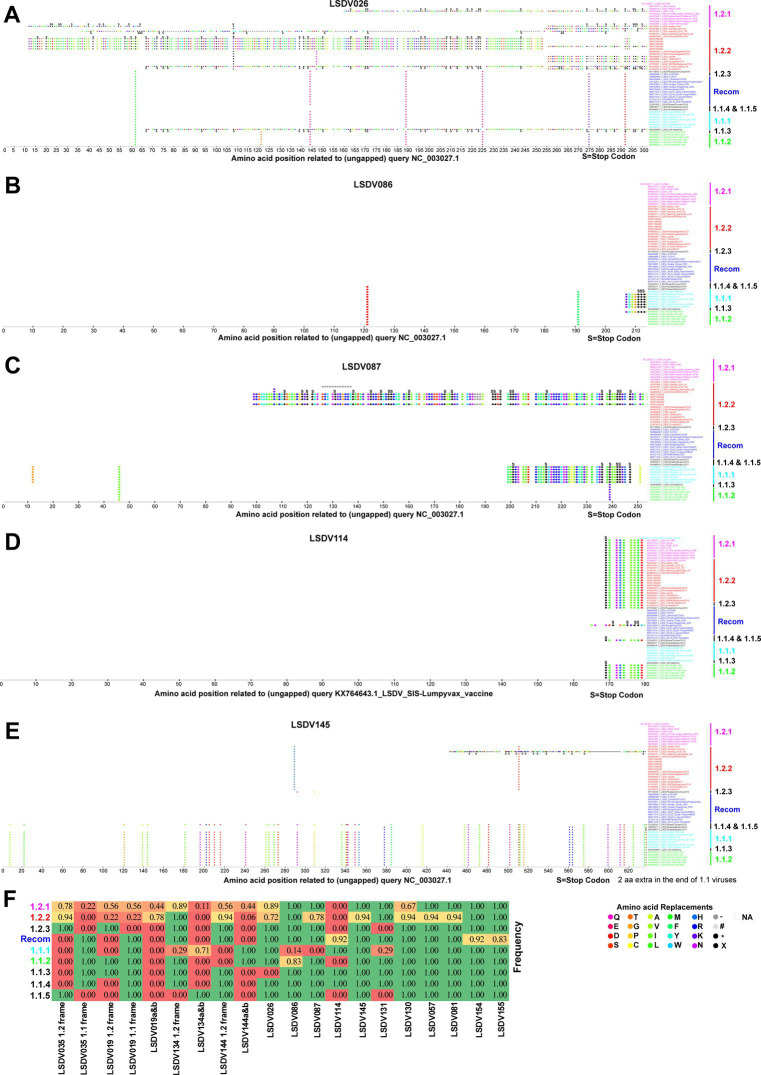
In-frame nonsense mutations in LSDV026, LSDV086, LSDV087, LSDV114, and LSDV145 genes. In-frame nonsense mutations in the gene **(A)** LSDV026; **(B)** LSDV086; **(C)** LSDV087; **(D)** LSDV114; and **(E)** LSDV145. Details of sequences used in this analysis are provided in Supplementary Data 1. **(F)** The table depicts the frequency of frameshift and in-frame nonsense mutations in different genes detected in the present study in each cluster.

In some viruses recently detected in the LSDV outbreak, LSDV019 and LSDV144 genes have been reported to be two fragments, LSDV019a, LSDV019b, and LSDV144a, LSDV144b, respectively, through frameshift mutations (Kumar et al., 2023), so we are interested in finding out which other genes have frameshift mutations and in-frame nonsense mutations among different clusters. In the present study, a frameshift mutation in LSDV035, LSDV019, LSDV134, and LSDV144 genes and in-frame non-sense mutations in LSDV026, LSDV086, LSDV087, LSDV114, LSDV130, LSDV131, LSDV145, LSDV154, LSDV155, LSDV057, and LSDV081 genes were revealed among different clusters (Figures 2–4; Supplementary Figure S5).

Remarkably, if the translation of the LSDV035 (putative RNA polymerase subunit-402 amino acid length) gene starts at the start codon frame and position of wild-type viruses, this gene is truncated in the vaccine, vaccine-derived, and recombinant viruses (Figure 2A). Whereas, if the LSDV035 gene starts translation in the start codon frame and position of vaccine, vaccine-derived, and recombinant viruses, this gene is truncated in wild-type viruses (Figure 2B). Also, it is significant that 32 amino acids “MFVLKLFNFNIYKNEFLVLLYLDFSINAKMENN” are extra at the N-terminal of the wild-type viruses (Figures 2A,B). In addition, OK422493.1/India/2019/Ranchi-1/P30, OK422494.1/India/2019/Ranchi-1/P50, and MT007951.1/Namibia/2016/10F viruses have LSDV035 gene truncated in both frames. It is also worth noting that the vaccine-derived virus OL542833.1/Russia/Tyumen/2019 follows the frame of wild-type viruses (Figures 2A,B).

Interestingly, if the translation of the LSDV019 (putative remodeling and stabilization of the host cytoskeleton and host immune evasion) gene is initiated in the start codon frame and position of wild-type viruses, this gene is truncated in viruses detected elsewhere except those detected in African countries (except AF409137.1/Neethling_Warmbaths/LW, and MW656253.1/280-KZN/RSA/2018 detected in Africa) (Figure 2C). Further, in this frame vaccine virus cluster 1.1.1 is also truncated; it is also worth noting that the SRR21590386 detected in India follows the frame of wild-type viruses (Figure 2C). On the other hand, if the LSDV019 gene starts the translation in the start codon frame and position of recombinant viruses, this gene is truncated in cluster-1.2 viruses detected in other places except the viruses detected in African countries (except AF409137.1/Neethling_Warmbaths/LW, and MW656253.1/280-KZN/RSA/2018 detected in Africa; SRR21590386). Also, it is significant that 129 amino acids “MTLKRYINKEYVEELKYMLLKNDYDRILIFTIGGISQTRKDIFEIS SNYFKKAIKKYSNEVKLPFKYKPFTYVLEYINTGYITLNSKNVVDIFAISNVIEIKFIM DACTDFMINYIDDSNCVDIFRRSY” are extra at the N-terminal of the wild-type viruses (Figures 2C,D). Also, the LSDV019 gene is translated in both frames in wild-type and vaccine-derived viruses found in African countries, but in vaccine cluster-1.1.1 and recombinant viruses, it is translated in only one frame and produces a protein with 129 amino acids less. Significantly, the LSDV019 gene has two fragments, LSDV019a, and LSDV019b, in wild-type cluster-1.2 viruses detected elsewhere, except for viruses detected in African countries (except AF409137.1/Neethling_Warmbaths/LW and MW656253.1/280-KZN/RSA/2018 detected in Africa) (Figures 2C–E).

Similarly, the LSDV134 (variola virus B22R-like protein) gene produces two fragments, LSDV134a and LSDV134b, in vaccine cluster-1.1.1 (except KX764644.1/Neethling-Herbivac/vaccine and MW656252.1/Haden/RSA/1954) and OL542833.1/Russia/Tyumen/2019 viruses (Figures 3A–C). LSDV134b is truncated in the KX683219.1/KSGP/0240 virus belonging to wild-type cluster-1.2.1 (Figures 3A–C). Additionally, the LSDV144 (putative remodeling and stabilization of the host cytoskeleton and host immune evasion) gene produces two fragments, LSDV144a, and LSDV144b, in vaccine cluster-1.1.1, vaccine-derived cluster 1.1.2 and wild-type cluster-1.2.1 viruses detected from India (Figures 3D,E).

Further, our analysis revealed that the LSDV026 gene was truncated by in-frame non-sense mutations in viruses belonging to wild-type cluster-1.2.2, except for viruses such as AF409137.1/Neethling-Warmbaths/LW, MW656253.1/280-KZN/RSA/2018, MW699032.1 /Russia/Dagestan/2015, MH893760.2/Russia/Dagestan/2015, and KY702007.1/SERBIA/Bujanovac/2016 (Figure 4A). The LSDV026 gene was truncated by in-frame non-sense mutations in MW631933.1_LSDV_LSD viruses belonging to wild-type cluster-1.2.1 and MW435866.1_LSDV_SA-Neethling viruses belonging to cluster-1.1.3 (Figure 4A). Similarly, the LSDV086 (similar to vaccinia virus strain Copenhagen D9R) gene was found to be truncated by in-frame non-sense mutations in viruses belonging to vaccine cluster-1.1.1 except MW656252.1_LSDV/Haden/RSA/195 virus (Figure 4B). The LSDV086 gene was truncated by in-frame non-sense mutations in MN636839.1_LSD-103-GP-RSA-1991 virus belonging to vaccine-derived (Figure 4B). Also, LSDV087 (similar to vaccinia virus strain Copenhagen D10R) gene has been truncated by in-frame non-sense mutations in viruses belonging to vaccine cluster-1.1.1 and SRR21590382, SRR21590384, SRR21590385, and SRR2159038 belonging to wild-type cluster-1.2.2 found in India (Figure 4C). Interestingly, the LSDV114 gene is truncated by in-frame non-sense mutations in viruses other than vaccine cluster-1.1.1, cluster-1.1.5, and recombinant clusters (except MW732649.1_LSDV/HongKong) (Figure 4D). Finally, the LSDV145 (ankyrin repeat protein) gene is truncated by in-frame non-sense mutations in viruses other than cluster-1.1 viruses (Figure 4E), and the LSDV131 (superoxide dismutase precursor) gene is truncated in the majority of the vaccine strains in the cluster-1.1.1 (Supplementary Figure S5A).

In addition, LSDV130 gene was truncated by in-frame non-sense mutations only in OK422492.1/Cattle/India/2019/Ranchi-1/P10, OK422493.1/India/2019/Ranchi-1/P30, OK422494.1/India/2019/Ranchi-1/P50 and OK318001.1/isolate-V28 viruses (Supplementary Figure S5B). Similarly, LSDV057, LSDV081, LSDV154, and LSDV155 genes were found to be truncated by in-frame non-sense mutations only in viruses MT007951.1/Namibia/2016/10F, MH893760.2/Russia/Dagestan/2015, OM984486.1/FJ2019, and OM793603.1/Russia/Khabarovsk/2020& MW732649.1/HongKong/2020 viruses, respectively (Supplementary Figures S5C–F).

It appears that none of the detected frameshift mutations and in-frame nonsense mutations (except LSDV114) are common to all viruses in a particular wild-type cluster-1.2.1, whereas these mutations are common among some of the viruses in different clusters of vaccine, vaccine-derived, and recombinant viruses (Figure 4F). A similar trend was observed in wild-type cluster-1.2.2 genes except for LSDV114, LSDV035, and LSDV019 (Figure 4F). Also, it is noteworthy that there were no detected frameshift mutations and in-frame nonsense mutations present only in all the viruses in the vaccine cluster-1.1.1 (Figure 4F). From these, since these frameshift and in-frame nonsense mutations are common among viruses in different clusters detected at various geographical locations at different times, these frameshift and in-frame nonsense mutations are likely caused by some common factors such as host adaptation, immune evasion, and recombination.

### Purifying selection is the dominant driver of LSDV evolution

**Figure 5:**
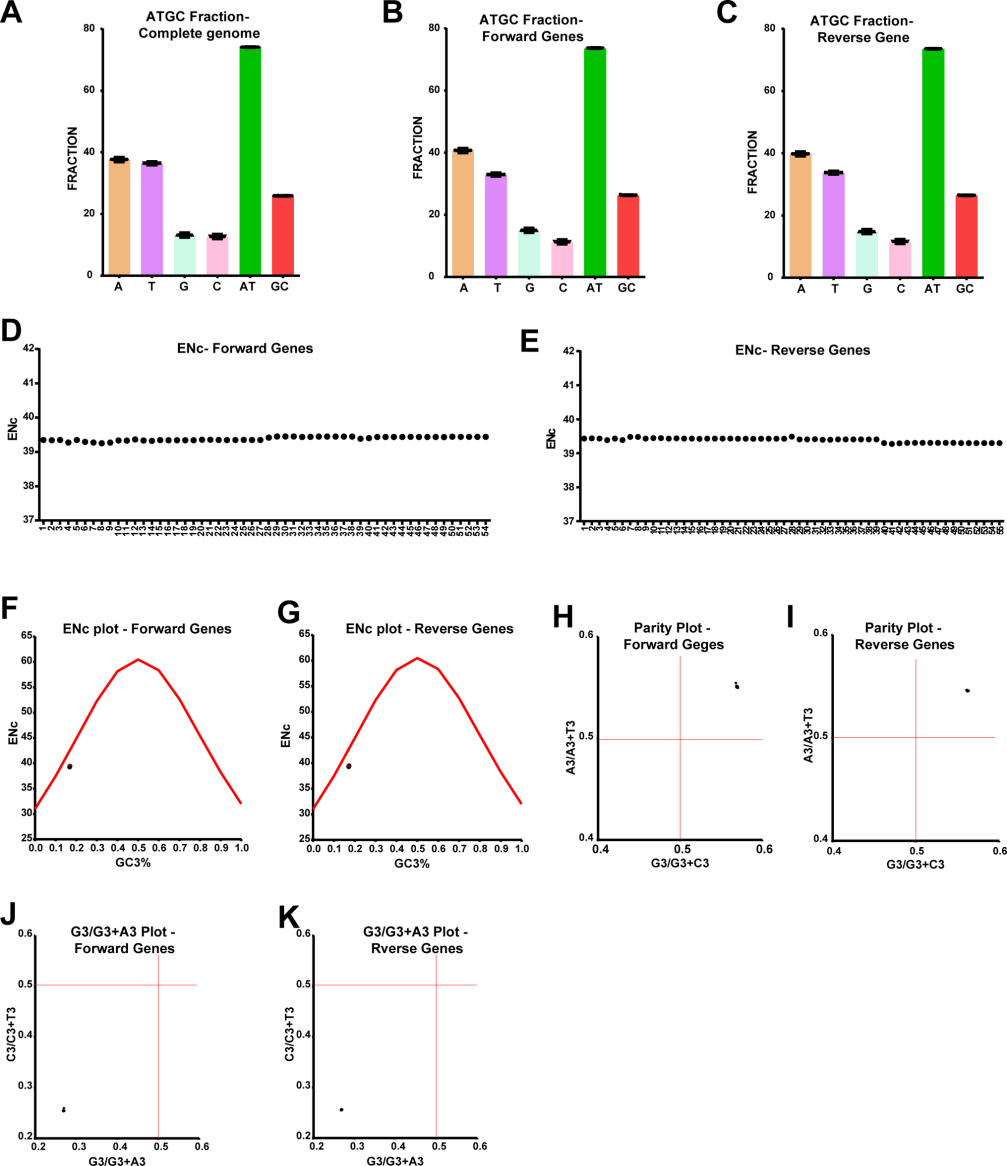
Host codons usage bias, selection pressure, codon third position CT-bias, and GA-bias in the coding regions of LSD viruses. **(A–C)** The graphs depict the nucleotide composition of LSD viruses, **(A)** at complete genome levels; **(B)** genes that are transcribing in the forward direction; and **(C)** genes that are transcribing in the reverse direction. **(D,E)** The graphs display the ENc values in the coding regions of LSD viruses, **(D)** genes that are transcribing in the forward direction; and **(E)** genes that are transcribing in the reverse direction. **(F,G)** The graphs illustrate the ENc-plot in the coding regions of LSD viruses, **(F)** genes that are transcribing in the forward direction, and **(G)** genes that are transcribing in the reverse direction. **(H,I)** The graphs show the parity-plot in the coding regions of LSD viruses, **(H)** genes that are transcribing in the forward direction; and **(I)** genes that are transcribing in the reverse direction. **(H,I)** The graphs depict the G3/G3 + A3-plot in the coding regions of LSD viruses, **(J)** genes that are transcribing in the forward direction; and **(K)** genes that are transcribing in the reverse direction. Details of sequences used in this analysis are provided in Supplementary Datas 2–4.

In the earlier sections, we analyzed differences in genes associated with frameshift mutation and in-frame nonsense mutations between different clusters of LSD viruses, so here we aimed to find out what differences exist in genes other than those described above. For this, we included genes that were not analyzed in the earlier section and also did not have open reading frames (ORF) overlaps; further, the genes that are transcribed in forward and reverse directions were analyzed separately in this study. First, we analyzed the nucleotide composition in the coding regions, and this analysis revealed that “AT” occupies around 75% of the nucleotides in genes transcribed in both forward and reverse directions, similar to the whole genome level (Figures 5A–C). From these, it is revealed that there is a bias in “AT” and “GC” in LSD viruses at the complete genome levels and the coding regions. Therefore, we were interested in whether this “AT” bias in coding regions could mediate codon usage bias among different clusters of LSD viruses. For this, we first performed an effective number of codons (ENc) analysis (Zhao et al., 2016; Wang et al., 2018; Desingu and Nagarajan, 2022; Desingu et al., 2022a,b), and this analysis revealed that ENc was around 39 of the genes transcribed in forward and reverse directions of all LSD viruses (Figures 5D,E). From these results, LSD viruses effectively use only 39 codons out of 61 codons in hosts to produce 20 amino acids, so it is clear that LSD viruses have a bias in using host codons. Further, in the analysis of ENc-GC3s plot (Wang et al., 2018; Tian et al., 2020; Desingu and Nagarajan, 2022; Desingu et al., 2022a,b), the genes transcribed in forward and reverse directions of LSD viruses fall slightly below the expected curve (Figures 5F,G), so it could be realized that selection pressure has a little more influence than mutation pressure in the evolution of these genes. Next, we performed PR2-bias analysis to detect AT bias and GC-bias (Tian et al., 2020; Desingu and Nagarajan, 2022; Desingu et al., 2022a,b), in which AT bias [A3/(A3 + T3)] was plotted against GC-bias [G3/(G3 + C3)]. In this PR2-bias analysis, since genes transcribed in the forward and reverse directions of LSD viruses are around 0.5 (Figures 5H,I), A-to-T and G-to-C bias do not significantly account for the bias in codon usage. Furthermore, CT bias [C3/(C3 + T3)] was plotted against GA-bias [G3/(G3 + A3)], and in the analysis conducted to detect CT bias and GA-bias, genes transcribed in forward and reverse directions of LSD viruses were around 0.25 (Figures 5J,K), suggests that C (25%) to T (75%) bias and G (25%) to A (75%) bias largely account for the bias in codon usage. Collectively, from the host codons usage bias, selection pressure, codon third position CT bias, and GA-bias in the coding regions of LSD viruses, it could be inferred that LSD viruses are more likely to mutate to adapt to the new host’s synonymous codon usage and achieve evolutionary development if they undergo host-jump or (or) pass through unnatural hosts for virus attenuation.

**Figure 6:**
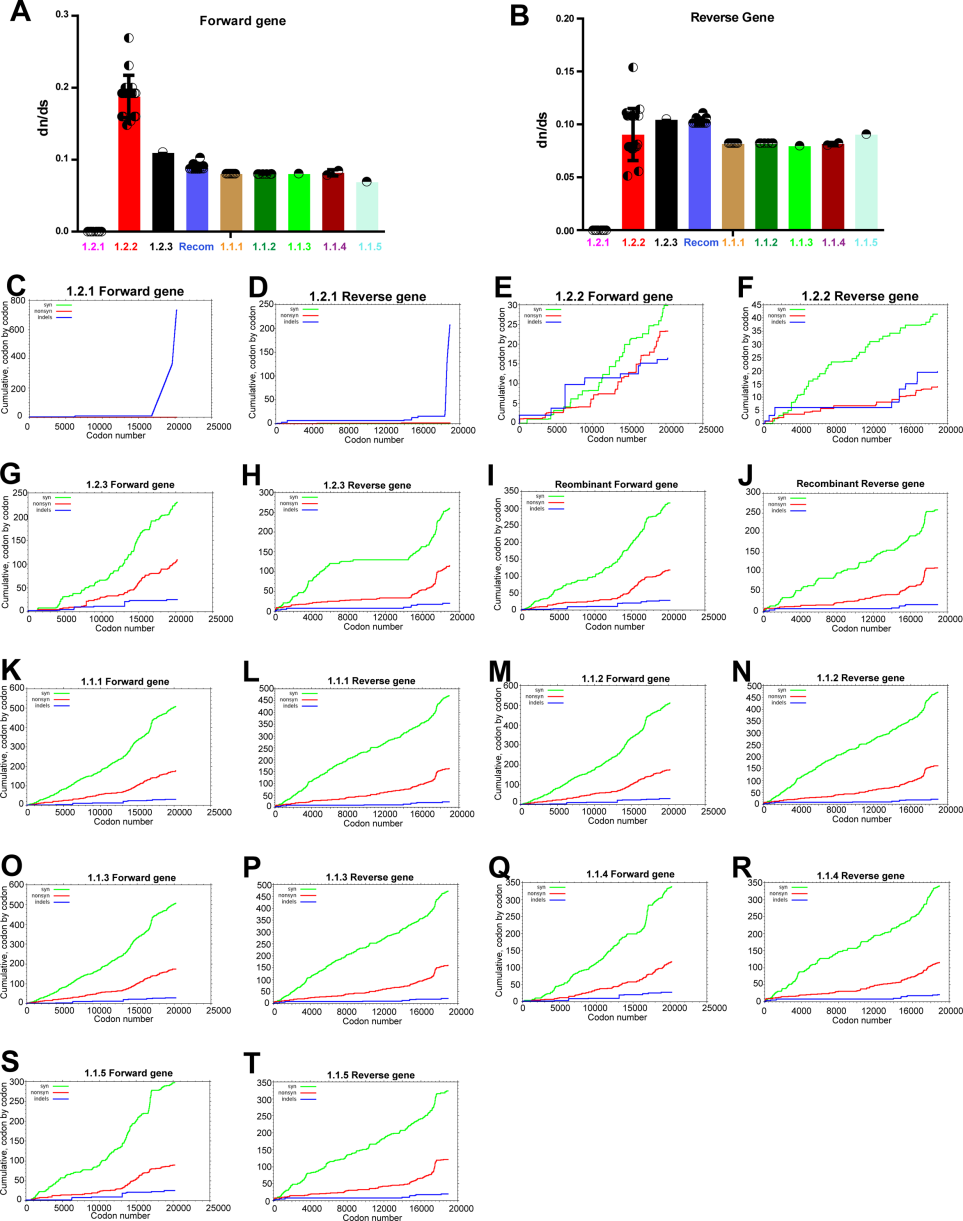
Purifying selection in the coding regions of LSD viruses. **(A,B)** The graphs depict the d*N*/d*S* ratio in the coding regions of different clusters of LSD viruses, **(A)** genes that are transcribing in the forward direction; and **(B)** genes that are transcribing in the reverse direction. The NC_003027.1 sequence was used as a reference in this analysis. **(C–T)** The graphs depict the cumulative d*N*/d*S* ratio in the coding regions of proteins from the center to ITR regions of different clusters of LSD viruses. The NC_003027.1 sequence was used as a reference in this analysis. Details of sequences used in this analysis are provided in Supplementary Datas 5 and 6. The forward and reverse directions for transcribing gene names and orders are presented in Supplementary Figures S3, S4.

After this, we were interested in finding out whether LSDV-vaccine strains attenuated by the passage in the unnatural host or the unnatural host’s cell culture have evolved to adapt to the usage of synonymous codons of the unnatural host. For this purpose, we performed a d*N*/d*S* analysis comparing LSDV wild-type NCBI reference strain NC_003027.1_LSDV_NI-2490 with viruses from other clusters. This d*N*/d*S* analysis revealed negative/purifying selection in genes transcribed in forward and reverse directions in clusters of vaccine, vaccine-derived, and recombinant viruses compared to wild-type (NC_003027.1) (Figures 6A,B). When comparing vaccine, vaccine-derived, and recombinant viruses with wild-type (NC_003027.1) d*N*/d*S* is around 0.1 (Figures 6A,B), it is clear that most of the total mutations in these viruses are synonymous codon mutations. Further, synonymous and nonsynonymous mutations in each cluster were subjected to in-depth analysis. In this analysis, it was revealed that the presence of synonymous and nonsynonymous mutations in genes transcribed in forward and reverse directions in LSD viruses was almost equal, and specifically, synonymous mutations were more abundant than nonsynonymous mutations in all clusters compared to wild-type (NC_003027.1) (Figures 6C–T). Also, it was revealed that synonymous and nonsynonymous mutations in vaccine and vaccine-derived viruses are around 500 and 175, respectively, whereas synonymous and nonsynonymous mutations in recombinant viruses are around 300 and 100, respectively (Figures 6C–T). In addition, these synonymous and nonsynonymous mutations can be seen to increase from central to terminal genes in genes that are transcribed in forward and reverse directions in LSD viruses (Figures 6C–T). Notably, genes in the terminal part of the genome of poxviruses generally have high genetic diversity, and these genes play an essential role in host range, host adaptation, evasion of the host immunity, pathogenicity, and virulence (Biswas et al., 2020; Senkevich et al., 2020, 2021; Brennan et al., 2022).

Next, we were interested in identifying nonsynonymous mutations in the genes transcribed in the forward and reverse directions of viruses in the vaccine, vaccine-derived, and recombinant clusters compared to the wild-type virus. In this analysis, nonsynonymous mutations increased from central to terminal genes in genes transcribed in forward and reverse directions of viruses in the vaccine, vaccine-derived, and recombinant clusters compared to wild-type viruses (Supplementary Figures S6, S7). Further, it was observed that the nonsynonymous mutations unique to the viruses in the recombinant cluster were not at a significant level and were a mixture of wild-type cluster-1.2.1 and vaccine cluster-1.1.1 (Supplementary Figures S6, S7). Also, since the nonsynonymous mutations found in the viruses in the wild-type cluster-1.2.2 are mainly absent in the viruses in the vaccine-derived and recombinant clusters (Supplementary Figures S6, S7), it could be realized that the viruses in the wild-type cluster-1.2.2 are evolving in a different direction from the viruses in the vaccine, vaccine-derived, and recombinant clusters.

Overall, it could be felt that the attenuated vaccine strains have evolved possibly through purifying selection for host adaptation by attaining the majority of mutations in synonymous codons as adapted to the codon usage of unnatural hosts. Also, viruses in wild-type cluster-1.2.2 have a purifying selection compared to wild-type cluster-1.2.1, and LSD viruses affect animals such as cattle, buffaloes, springbok, impala and giraffe (Young et al., 1970; Le Goff et al., 2009; Namazi and Khodakaram Tafti, 2021); this purifying selection suggests that possibly host adaptation has resulted in the majority of mutations in synonymous codons adapted to the codon usage of these or other animal hosts.

### APOBEC editing is the dominant driver of LSDV evolution

**Figure 7:**
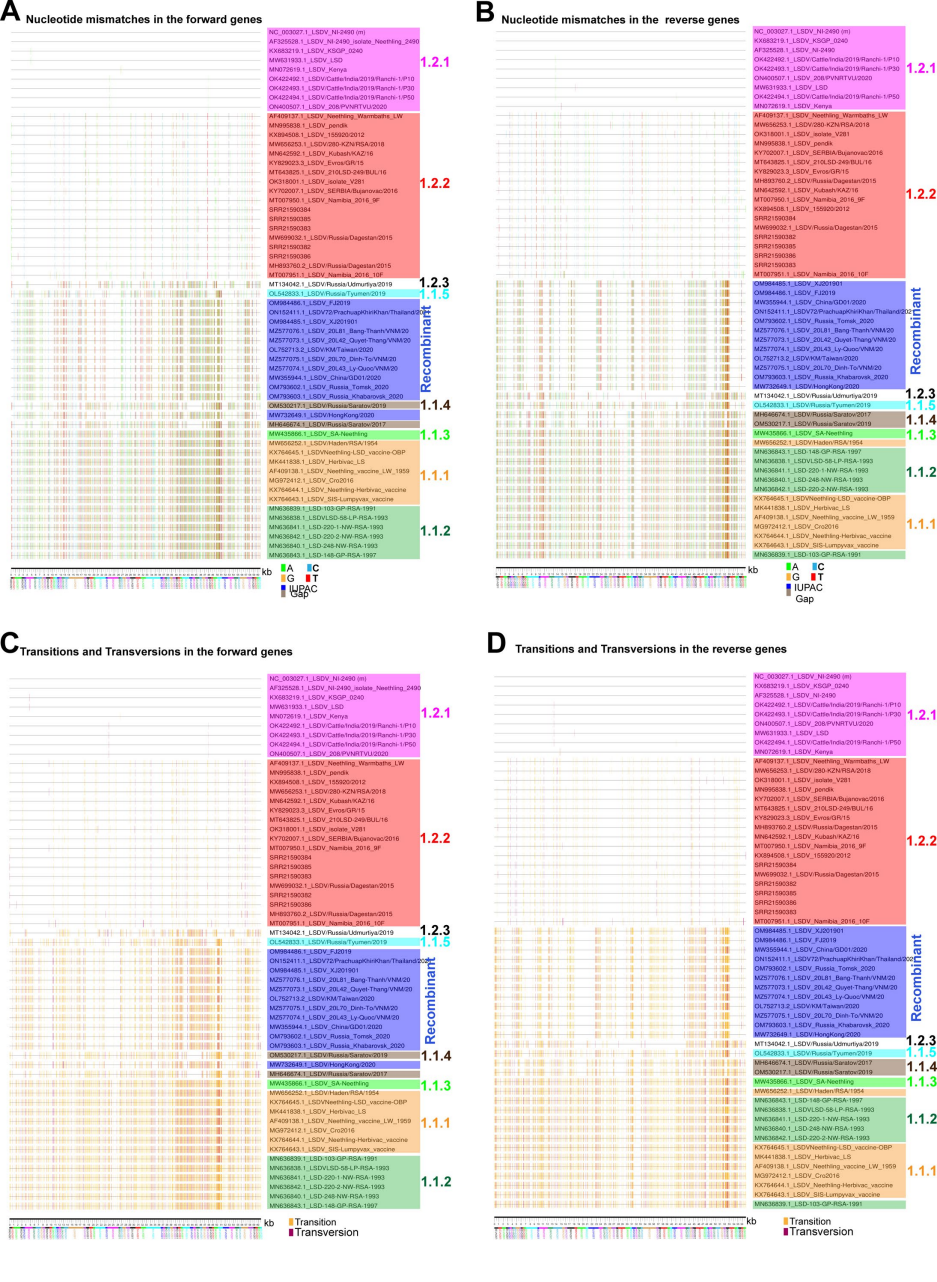
Nucleotide diversity in the coding regions of LSD viruses. **(A,B)** Nucleotide mismatches in the coding regions of LSD viruses were visualized; **(A)** genes that are transcribing in the forward direction and **(B)** genes that are transcribing in the reverse direction. The NC_003027.1 sequence was used as a reference in this analysis. **(C,D)** Transitions and transversions mutations in the coding regions of LSD viruses were visualized, **(C)** genes that are transcribing in the forward direction; and **(D)** genes that are transcribing in the reverse direction. The NC_003027.1 sequence was used as a reference in this analysis.

**Figure 8:**
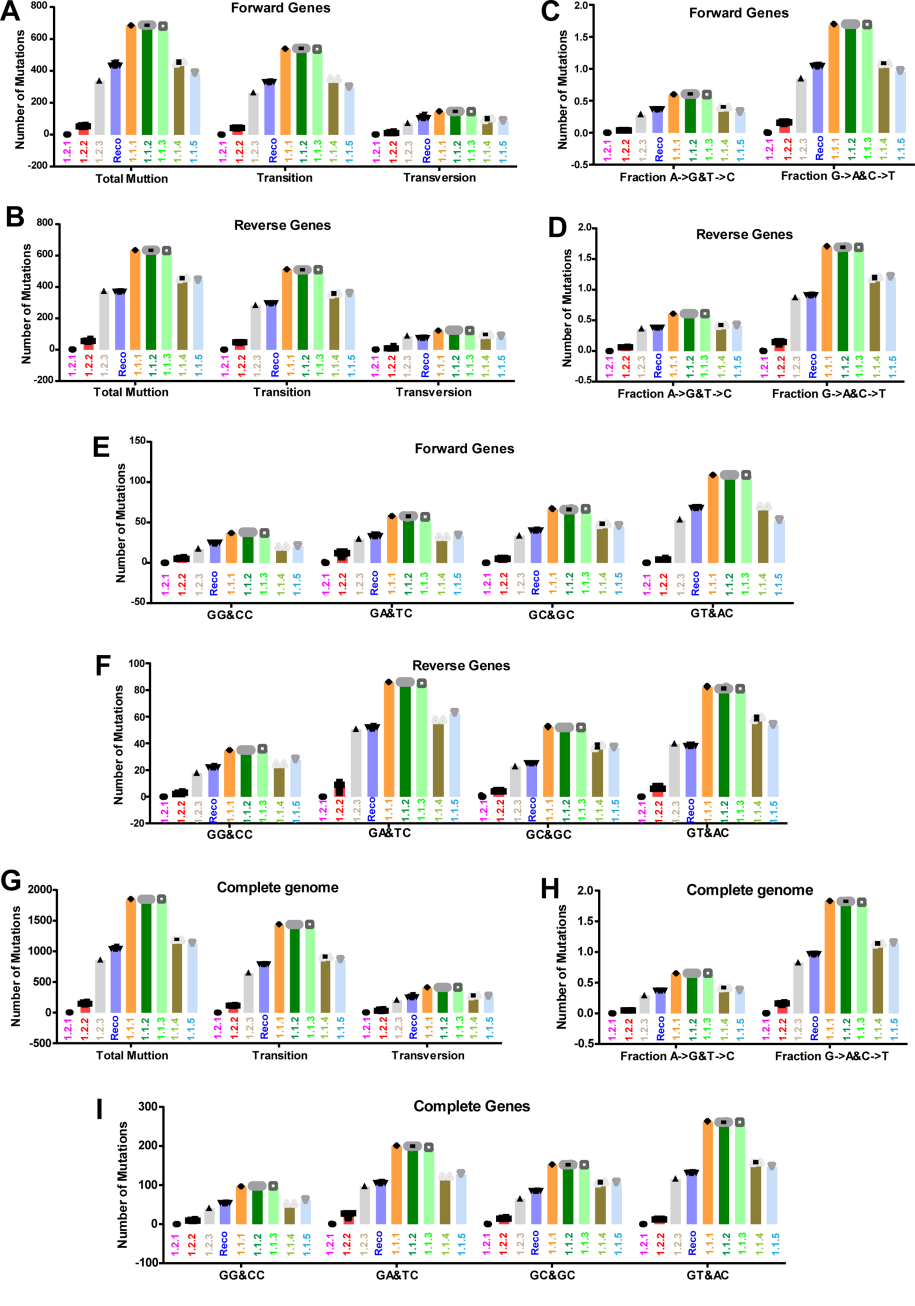
APOBEC editing at the coding regions and complete genome sequences level of LSD viruses. **(A,B)** The graphs depict the transitions and transversion mutations in the coding regions of different clusters of LSD viruses, **(A)** genes that are transcribing in the forward direction; and **(B)** genes that are transcribing in the reverse direction. The NC_003027.1 sequence was used as a reference in this analysis. **(C,D)** The graphs illustrate the Fraction A > G & T > C and Fraction G > A & C > T mutations in the coding regions of different clusters of LSD viruses, **(C)** genes that are transcribing in the forward direction; and **(D)** genes that are transcribing in the reverse direction. The NC_003027.1 sequence was used as a reference in this analysis. **(E,F)** The graphs elucidate the APOBEC motif mutations in the coding regions of different clusters of LSD viruses, **(E)** genes that are transcribing in the forward direction; and **(F)** genes that are transcribing in the reverse direction. The NC_003027.1 sequence was used as a reference in this analysis. **(G)** The graphs depict the transitions and transversion mutations at the complete genome levels of different clusters of LSD viruses. The NC_003027.1 sequence was used as a reference in this analysis. **(H)** The graphs illustrate the fraction A > G & T > C and fraction G > A & C > T mutations at the complete genome levels of different clusters of LSD viruses. The NC_003027.1 sequence was used as a reference in this analysis. **(I)** The graphs elucidate the APOBEC motif mutations at the complete genome levels of different clusters of LSD viruses. The NC_003027.1 sequence was used as a reference in this analysis. Details of sequences used in this analysis are provided in Supplementary Datas 7–9.

In the previous sections, in the attenuation and evolution of LSD viruses, mostly synonymous codons are evolved, and CT-bias and GA-bias are present in the third nucleotide position of codons, so here we are interested in finding out the mechanism by which such mutations are acquired. We investigated the nucleotide mismatches in genes transcribed in forward and reverse directions of LSD viruses in different clusters compared to wild-type NCBI reference strain NC_003027.1_LSDV_NI-2490. In this analysis, it can be realized that there are more nucleotide mismatches in the vaccine and vaccine-derived clusters, and these nucleotide mismatches are increasing from the central part toward the terminal part of the genes that are transcribed in the forward and reverse directions of LSD viruses (Figures 7A,B). Further, our analysis revealed that most of these nucleotide mismatches are silent mutations (Supplementary Figures S8A,B) and are caused by transition mutations (Figures 7C,D). Also, it is of increasing importance that around 80% of the mutations in genes transcribed in forward and reverse directions of LSD viruses of all clusters compared to wild-type (NC_003027.1) are transition mutations (Figures 8A,B). Furthermore, our results show that the G → A & C → T transition mutations fraction is around three times higher than the A → G & T → C transition mutations fraction in genes transcribed in forward and reverse directions of LSD viruses with all clusters compared to wild-type (NC_003027.1) (Figures 8C,D). Interestingly, G → A (or) C → T mutations can be generated by the host’s APOBEC enzymes. It is noteworthy that these APOBEC enzymes play an essential role in the restriction of retroviruses (HIV), DNA viruses such as monkeypox virus (Mpoxv), hepatitis B virus (HBV), and human papillomavirus (HPV) (Bonvin et al., 2006; Bulliard et al., 2011; Warren et al., 2015; Gigante et al., 2022; Isidro et al., 2022; Pecori et al., 2022). Since DNA editing by APOBEC enzymes is based on TC > TT (or) GA > AA, GG > AG (or) CC > CT, and AC > AA (or) GT > AT motifs (Gigante et al., 2022; Isidro et al., 2022; Pecori et al., 2022), we were interested in finding out what motif mutations are present in genes transcribed in forward and reverse directions of viruses in all clusters compared to wild-type (NC_003027.1). Genes transcribed in the forward directions of viruses in the vaccine, vaccine-derived, and recombinant clusters revealed a higher abundance of AC > AA & GT > AT motif mutations compared to wild-type (NC_003027.1) (Figure 8E; Supplementary Figure S9A). On the other hand, AC > AA & GT > AT and TC > TT & GA > AA motif mutations were found to be almost higher abundance in genes transcribed in reverse directions of viruses in the vaccine, vaccine-derived, and recombinant clusters compared to wild-type (NC_003027.1) (Figure 8F; Supplementary Figure S9B). After this, we were interested in finding out how the mutations created by these APOBEC enzymes compared to the wild-type (NC_003027.1) at the complete genome level of the viruses in the vaccine, vaccine-derived, and recombinant clusters. This analysis revealed that almost 80% of the mutations in the viruses in the vaccine, vaccine-derived, and recombinant clusters were transition mutations compared to the wild-type (NC_003027.1) at the whole genome level (Figure 8G), as in the coding regions (Figures 8A,B). Also, as in the coding regions, it was revealed that the G → A & C → T transition mutations fraction was almost three times higher than the A → G & T → C transition mutations fraction in the viruses in the vaccine, vaccine-derived, and recombinant clusters when compared to the wild-type (NC_003027.1) at the complete genome level (Figure 8H), and AC > AA & GT > AT and TC > TT & GA > AA motif mutations were also found to be more prevalent (Figure 8I; Supplementary Figure S9C). Interestingly, it is noteworthy that AC > AA & GT > AT motif mutations are edited by APOBEC1 enzyme present in tetrapod to humans, whereas TC > TT & GA > AA, GG > AG & CC > CT motif mutations are edited by APOBEC3 enzyme present in placental mammals (Gigante et al., 2022; Pecori et al., 2022). Overall, the viruses in the vaccine clusters have a large number of transition mutations at the complete genome and coding region level compared to the wild-type virus, and the G → A & C → T transition mutations fraction is higher in these transition mutations, and the G → A & C → T mutations are in motif mutations that are genome editing by host APOBEC enzymes. Also, since transition mutations in coding regions are mostly silent mutations, it is revealed that APOBEC enzymes are the dominant driver in the evolution of host codon usage adaptation of these vaccine viruses.

## Discussion

As the monkeypox virus in humans has caused outbreaks in non-endemic countries, recently, the lumpy skin disease virus (LSDV) Poxvirus in ruminants has caused outbreaks in non-endemic countries. However, the role of evolutionary drivers and genetic variations (frameshift and in-frame nonsense mutations) of LSDVs, which cause outbreaks in cattle in various countries and cause substantial economic losses, are still largely unknown. Possibly, LSDV outbreaks spread in new regions by the movements of infected cattle or vectors. As the Monkeypox virus spread to non-endemic countries from 2017 onwards (Yinka-Ogunleye et al., 2019; Mauldin et al., 2022; Desingu et al., 2022a,b), LSDV started spreading in the non-endemic countries (Krotova et al., 2022a,b). On the other hand, APOBEC3 mutations were found to be enriched only in clade-IIb viruses when compared to the most recent common ancestors of viruses within the clade and considered as the major driver of human adaptation-mediated microevolution (Gigante et al., 2022; Isidro et al., 2022). The present study sheds light on the microevolution of APOBEC editing, purifying selection, frameshift, and in-frame nonsense mutations in LSD viruses.

Homologous vaccines containing the Neethling strain have been attenuated by a very high number of passages in cell cultures and chicken eggs (Kitching, 2003; Vandenbussche et al., 2022), and studies have shown that this vaccine generally induces good protection and mild to negligible adverse reactions in cattle (Klement et al., 2020; Morgenstern and Klement, 2020; Bamouh et al., 2021; Haegeman et al., 2021). Studies reported that the Kenyan sheep and goat pox (KSGP) strains vaccine contains lumpy skin disease viruses (Tuppurainen et al., 2014; Vandenbussche et al., 2016, 2022), and this vaccine has been reported to cause clinical signs in vaccinated cattle (Bamouh et al., 2021; Tuppurainen et al., 2021), this may be due to the lower number of passages to attenuate the virus (Vandenbussche et al., 2022). Recombinant LSDVs with genetic signatures of Neethling vaccine strain and KSGP vaccine strains have recently been reported in several studies (Sprygin et al., 2018a,b; Mathijs et al., 2021; Flannery et al., 2022; Huang et al., 2022; Ma et al., 2022). Further, a study using short-read next-generation sequencing methods reported that the KSGP vaccine contains lumpy skin disease viruses such as the Neethling-like LSDV vaccine strain, KSGP-like LSDV vaccine strain, and almost identical recombinant LSDV strains detected in the field outbreaks, suggesting recombinant LSDVs may be originated by recombination of the Neethling-like LSDV vaccine strain and KSGP-like LSDV vaccine strains in the vaccine (Vandenbussche et al., 2022).

Generally, LSD viruses are attenuated by serial passages in the unnatural host or unnatural host cells such as chicken eggs, rabbit kidney cells, and lamb kidney cells (Wallace and Viljoen, 2005; Tuppurainen et al., 2021). Viruses in general, are transformed into vaccine strains by passages in the unnatural host or unnatural host cells. In the process of attenuation, these viruses mostly undergo host adaptation evolution and become attenuation. In such evolution for host adaptation, mutations are adopted in synonyms codons to adapt to host codon usage bias. Non-synonymous mutations are adopted for virus-host cell entry, replication, and host immune evasion (Desingu and Nagarajan, 2022; Desingu et al., 2022a,b). In general, ENc values <35 are considered to have high codon bias, and >50 to indicate general random codon usage (Sheikh et al., 2020; Desingu and Nagarajan, 2022; Li et al., 2022). In the present study, we observed the ENc value around 39 in LSD viruses suggesting moderate codon use bias in the LSD viruses, and this ENc value is possibly associated with the limited host tropism of LSDVs. Interestingly, the present study revealed that compared to wild-type virus, attenuated vaccine strains have more transition mutations, G → A & C → T transition mutations fraction is greater than A → G & T → C, and G → A & C → T are APOBEC editing motif mutations. Further, we observed negative/purifying selection in genes transcribed in forward and reverse directions in clusters of vaccine, vaccine-derived, and recombinant viruses in the d*N*/d*S* analysis. Consistent with this, we also noticed the abundance of synonymous codon mutations in attenuated vaccine strains compared to wild-type viruses revealed moderate selection pressure for host codon usage bias. In addition, the present study identified the frameshift mutations and in-frame nonsense mutations in distinct ORF disturbances in different clusters will help to understand the pathogenic importance of these ORFs in LSD viruses by future experimental studies and also monitor the epidemiological spread of viruses. Further, we observed that even though the viruses causing the outbreaks are grouped in a specific cluster at the whole genome level, they have attained ORF disturbance similar to other clusters. From these, it could be realized that the attenuated vaccine strains and evolution of field strains could have been transformed into vaccine strains and mutant strains, respectively, through gene deletion, host selection pressure, purifying selection, and APOBEC editing.

In conclusion, it is revealed that LSD viruses have achieved microevolution through host selection pressure, purifying selection, and APOBEC editing. There are unique frameshift and in-frame nonsense mutations in the specific genes of most viruses in each cluster. Also, it has come to light that despite being grouped in a specific cluster at the complete genome level, some genes have frameshift and in-frame nonsense mutations similar to those in other clusters and have mutated into viruses, causing outbreaks in different geographical regions. The findings in the present study are expected to help in the virus pathogenesis studies of disturbed ORFs in LSD viruses, and monitoring the epidemiological spread of viruses and their genetic variants.

## Data availability statement

The original contributions presented in the study are included in the article/Supplementary material, further inquiries can be directed to the corresponding author.

## Author contributions

PD conceived, designed the study, performed the data analysis, data interpretation, participated in the first draft writing, review and editing, generation of the final version of the manuscript, and acquisition of funding. TR assisted PD in the data analysis. KN, NS, and TR are involved in review and editing. All authors contributed to the article and approved the submitted version.

## Footnotes

^1^ https://mafft.cbrc.jp/alignment/server/

^2^ https://www.hiv.lanl.gov/content/index

## Supplementary material

Supplementary Figure S1 A recombination detection program (RDP) analysis based on potential recombination events is depicted for each virus.

Supplementary Figure S2 Graphical representation of potential recombination events in **(A)** OM984485.1/LSDV/XJ201901; **(B)** OM984486.1/LSDV/FJ2019; **(C)** MW355944.1/LSDV/ China/GD01/2020; **(D)** MZ577073.1/LSDV/20L42/Quyet-Thang/VNM/20; **(E)** MZ577074.1/LSDV_20L43/Ly-Quoc/VNM/20; **(F)** MZ577076.1_LSDV_20L81_Bang-Thanh/VNM/20; **(G)** MZ577075.1_LSDV_20L70_Dinh-To/VNM/20; and **(H)** OL752713.2/LSDV/KM/Taiwan/2020.

Supplementary Figure S3 Graphical representation of potential recombination events in **(A)** OM793602.1_LSDV_Russia_Tomsk_2020; **(B)** ON152411.1_LSDV72/PrachuapKhiriKhan/Thailand/2021; **(C)** OM793603.1_LSDV_Russia_Khabarovsk_2020; **(D)** MW732649.1/ LSDV/HongKong/2020; **(E)** MT134042.1_LSDV/Russia/Udmurtiya/2019; **(F)** OL542833.1/ LSDV/Russia/Tyumen/2019; **(G)** OM530217.1_LSDV/Russia/Saratov/2019; and **(H)** MH646674.1/LSDV/Russia/Saratov/2017.

Supplementary Figure S4 Transitions and Transversion mutations at the complete genome levels of different clusters of LSD viruses were visualized, and the NC_003027.1 sequence was used as a reference in this analysis.

Supplementary Figure S5 In-frame nonsense mutations in LSDV131, LSDV130, LSDV057, LSDV081, LSDV154 and LSDV155 genes. In-frame nonsense mutations in the gene **(A)** LSDV131; **(B)** LSDV130; **(C)** LSDV057; **(D)** LSDV081; **(E)** LSDV154 and **(F)** LSDV155. Details of sequences used in this analysis are provided in Supplementary Data 1.

Supplementary Figure S6 The amino acid mutations in the forward direction transcribe genes coding regions from the center to ITR regions of different clusters of LSD viruses. The NC_003027.1 sequence was used as a reference in this analysis.

Supplementary Figure S7 The amino acid mutations in the reverse direction transcribe genes coding regions from the center to ITR regions of different clusters of LSD viruses. The NC_003027.1 sequence was used as a reference in this analysis.

Supplementary Figure S8 **(A,B)** Silent and Non-silent mutations in the coding regions of LSD viruses were visualized, **(A)** genes that are transcribing in the forward direction and **(B)** genes that are transcribing in the reverse direction. The NC_003027.1 sequence was used as a reference in this analysis.

Supplementary Figure S9 The APOBEC motif mutations in the different clusters of LSD viruses were visualized, **(A)** genes that are transcribing in the forward direction, **(B)** genes that are transcribing in the reverse direction, and **(C)** at the complete genome levels. The NC_003027.1 sequence was used as a reference in this analysis.

Supplementary Figure S10 **(A–D)** The Plot LAST hits analysis shows the OK422492.1/India/2019/Ranchi-1/P10, OK422493.1//India/2019/Ranchi-1/P3, and ON400507.1/208/PVNRTVU/202 sequences were submitted as reverse complement compared to NCBI reference sequences NC_003027.1_LSDV_NI-249.

## References

[ref1] Abutarbush S. M. Ababneh M. M. Al Zoubi I. G. Al Sheyab O. M. Al Zoubi M. G. Alekish M. O.. (2015). Lumpy skin disease in Jordan: disease emergence, clinical signs, complications and preliminary-associated economic losses. Transbound. Emerg. Dis. 62, 549–554. doi: 10.1111/tbed.12177 24148185

[ref2] Acharya K. P. Subedi D. (2020). First outbreak of lumpy skin disease in Nepal. Transbound. Emerg. Dis. 67, 2280–2281. doi: 10.1111/tbed.13815, PMID: 32889764

[ref3] Badhy S. C. Chowdhury M. G. A. Settypalli T. B. K. Cattoli G. Lamien C. E. Fakir M. A. U.. (2021). Molecular characterization of lumpy skin disease virus (LSDV) emerged in Bangladesh reveals unique genetic features compared to contemporary field strains. BMC Vet. Res. 17:61. doi: 10.1186/s12917-021-02751-x, PMID: 33514360 PMC7844896

[ref4] Bamouh Z. Hamdi J. Fellahi S. Khayi S. Jazouli M. Tadlaoui K. O.. (2021). Investigation of post vaccination reactions of two live attenuated vaccines against lumpy skin disease of cattle. Vaccines 9. doi: 10.3390/vaccines9060621, PMID: 34201339 PMC8226854

[ref5] Bankevich A. Nurk S. Antipov D. Gurevich A. A. Dvorkin M. Kulikov A. S.. (2012). SPAdes: a new genome assembly algorithm and its applications to single-cell sequencing. J. Comput. Biol. 19, 455–477. doi: 10.1089/cmb.2012.0021, PMID: 22506599 PMC3342519

[ref6] Bhatt L. Bhoyar R. C. Jolly B. Israni R. Vignesh H. Scaria V.. (2023). The genome sequence of lumpy skin disease virus from an outbreak in India suggests a distinct lineage of the virus. Arch. Virol. 168:81. doi: 10.1007/s00705-023-05705-w 36740645

[ref7] Biswas S. Noyce R. S. Babiuk L. A. Lung O. Bulach D. M. Bowden T. R.. (2020). Extended sequencing of vaccine and wild-type capripoxvirus isolates provides insights into genes modulating virulence and host range. Transbound. Emerg. Dis. 67, 80–97. doi: 10.1111/tbed.13322, PMID: 31379093

[ref8] Bolger A. M. Lohse M. Usadel B. (2014). Trimmomatic: a flexible trimmer for Illumina sequence data. Bioinformatics 30, 2114–2120. doi: 10.1093/bioinformatics/btu170, PMID: 24695404 PMC4103590

[ref9] Bonvin M. Achermann F. Greeve I. Stroka D. Keogh A. Inderbitzin D.. (2006). Interferon-inducible expression of APOBEC3 editing enzymes in human hepatocytes and inhibition of hepatitis B virus replication. Hepatology 43, 1364–1374. doi: 10.1002/hep.21187 16729314

[ref10] Brennan G. Stoian A. M. M. Yu H. Rahman M. J. Banerjee S. Stroup J. N.. (2022). Molecular mechanisms of poxvirus evolution. mBio 14:e0152622. doi: 10.1128/mbio.01526-22, PMID: 36515529 PMC9973261

[ref11] Buchfink B. Xie C. Huson D. H. (2015). Fast and sensitive protein alignment using DIAMOND. Nat. Methods 12, 59–60. doi: 10.1038/nmeth.3176, PMID: 25402007

[ref12] Bulliard Y. Narvaiza I. Bertero A. Peddi S. Röhrig U. F. Ortiz M.. (2011). Structure-function analyses point to a polynucleotide-accommodating groove essential for APOBEC3A restriction activities. J. Virol. 85, 1765–1776. doi: 10.1128/JVI.01651-10, PMID: 21123384 PMC3028873

[ref13] Chibssa T. R. Sombo M. Lichoti J. K. Adam T. I. B. Liu Y. Elraouf Y. A.. (2021). Molecular analysis of east African lumpy skin disease viruses reveals a mixed isolate with features of both vaccine and field isolates. Microorganisms 9. doi: 10.3390/microorganisms9061142, PMID: 34073392 PMC8229927

[ref14] Deforche K. (2017). An alignment method for nucleic acid sequences against annotated genomes. bioRxiv 200394. doi: 10.1101/200394

[ref15] Desingu P. A. Nagarajan K. (2022). Genetic diversity and characterization of circular replication (rep)-encoding single-stranded (CRESS) DNA viruses. Microbiol. Spectr. 10:e0105722. doi: 10.1128/spectrum.01057-22, PMID: 36346238 PMC9769708

[ref16] Desingu P. A. Nagarajan K. Dhama K. (2022a). SARS-CoV-2 gained a novel spike protein S1-N-terminal domain (S1-NTD). Environ. Res. 211:113047. doi: 10.1016/j.envres.2022.113047, PMID: 35292244 PMC8917877

[ref17] Desingu P. A. Rubeni T. P. Sundaresan N. R. (2022b). Evolution of monkeypox virus from 2017 to 2022: in the light of point mutations. Front. Microbiol. 13:1037598. doi: 10.3389/fmicb.2022.1037598, PMID: 36590408 PMC9795006

[ref18] Flannery J. Shih B. Haga I. R. Ashby M. Corla A. King S.. (2022). A novel strain of lumpy skin disease virus causes clinical disease in cattle in Hong Kong. Transbound. Emerg. Dis. 69, e336–e343. doi: 10.1111/tbed.14304, PMID: 34448540

[ref19] Ganeshan S. Dickover R. E. Korber B. T. Bryson Y. J. Wolinsky S. M. (1997). Human immunodeficiency virus type 1 genetic evolution in children with different rates of development of disease. J. Virol. 71, 663–677. doi: 10.1128/JVI.71.1.663-677.1997, PMID: 8985398 PMC191099

[ref20] Gigante C. M. Korber B. Seabolt M. H. Wilkins K. Davidson W. Rao A. K.. (2022). Multiple lineages of monkeypox virus detected in the United States, 2021–2022. Science 378, 560–565. doi: 10.1126/science.add4153, PMID: 36264825 PMC10258808

[ref21] Haegeman A. de Leeuw I. Mostin L. Campe W. V. Aerts L. Venter E.. (2021). Comparative evaluation of lumpy skin disease virus-based live attenuated vaccines. Vaccines 9. doi: 10.3390/vaccines9050473, PMID: 34066658 PMC8151199

[ref22] Huang C. W. Ting L. J. Liu Y. P. Lin Y. J. Lee F. Chiou C. J. (2022). Complete coding sequence of lumpy skin disease virus isolated from Kinmen Island, Taiwan, in 2020. Microbiol. Resour. Announc. 11:e0120421. doi: 10.1128/mra.01204-21, PMID: 35297682 PMC9022544

[ref23] Isidro J. Borges V. Pinto M. Sobral D. Santos J. D. Nunes A.. (2022). Phylogenomic characterization and signs of microevolution in the 2022 multi-country outbreak of monkeypox virus. Nat. Med. 28, 1569–1572. doi: 10.1038/s41591-022-01907-y, PMID: 35750157 PMC9388373

[ref24] Katoh K. Frith M. C. (2012). Adding unaligned sequences into an existing alignment using MAFFT and LAST. Bioinformatics 28, 3144–3146. doi: 10.1093/bioinformatics/bts578 23023983 PMC3516148

[ref25] Katoh K. Rozewicki J. Yamada K. D. (2019). MAFFT online service: multiple sequence alignment, interactive sequence choice and visualization. Brief. Bioinform. 20, 1160–1166. doi: 10.1093/bib/bbx108, PMID: 28968734 PMC6781576

[ref26] Katoh K. Standley D. M. (2013). MAFFT multiple sequence alignment software version 7: improvements in performance and usability. Mol. Biol. Evol. 30, 772–780. doi: 10.1093/molbev/mst010, PMID: 23329690 PMC3603318

[ref27] Keele B. F. Giorgi E. E. Salazar-Gonzalez J. F. Decker J. M. Pham K. T. Salazar M. G.. (2008). Identification and characterization of transmitted and early founder virus envelopes in primary HIV-1 infection. Proc. Natl. Acad. Sci. U. S. A. 105, 7552–7557. doi: 10.1073/pnas.0802203105 18490657 PMC2387184

[ref28] Kitching R. P. (2003). Vaccines for lumpy skin disease, sheep pox and goat pox. Dev. Biol. 114, 161–167. 14677686

[ref29] Klement E. Broglia A. Antoniou S. E. Tsiamadis V. Plevraki E. Petrović T.. (2020). Neethling vaccine proved highly effective in controlling lumpy skin disease epidemics in the Balkans. Prev. Vet. Med. 181:104595. doi: 10.1016/j.prevetmed.2018.12.001, PMID: 30553537

[ref30] Korber B. (2000). “HIV signature and sequence variation analysis” in Computational analysis of HIV molecular sequences. eds. Rodrigo A. G. Learn G. H. (Dordrecht: Kluwer Academic Publishers), 55–72.

[ref31] Krotova A. Byadovskaya O. Shumilova I. van Schalkwyk A. Sprygin A. (2022b). An in-depth bioinformatic analysis of the novel recombinant lumpy skin disease virus strains: from unique patterns to established lineage. BMC Genomics 23:396. doi: 10.1186/s12864-022-08639-w, PMID: 35610557 PMC9131581

[ref32] Krotova A. Byadovskaya O. Shumilova I. Zinyakov N. van Schalkwyk A. Sprygin A. (2022a). Molecular characterization of a novel recombinant lumpy skin disease virus isolated during an outbreak in Tyumen, Russia, in 2019. Transbound. Emerg. Dis. 69, e2312–e2317. doi: 10.1111/tbed.14574 35488786

[ref33] Kumar S. M. (2011). An outbreak of lumpy skin disease in a Holstein dairy herd in Oman: a clinical report. Asian J. Anim. Vet. Adv. 6, 851–859. doi: 10.3923/ajava.2011.851.859

[ref34] Kumar S. Stecher G. Tamura K. (2016). MEGA7: molecular evolutionary genetics analysis version 7.0 for bigger datasets. Mol. Biol. Evol. 33, 1870–1874. doi: 10.1093/molbev/msw054, PMID: 27004904 PMC8210823

[ref35] Kumar N. Tripathi B. N. (2022). A serious skin virus epidemic sweeping through the Indian subcontinent is a threat to the livelihood of farmers. Virulence 13, 1943–1944. doi: 10.1080/21505594.2022.2141971, PMID: 36320159 PMC9635543

[ref36] Kumar A. Venkatesan G. Kushwaha A. Poulinlu G. Saha T. Ramakrishnan M. A.. (2023). Genomic characterization of lumpy skin disease virus (LSDV) from India: circulation of Kenyan-like LSDV strains with unique kelch-like proteins. Acta Trop. 241:106838. doi: 10.1016/j.actatropica.2023.106838, PMID: 36796571

[ref37] Le Goff C. Lamien C. E. Fakhfakh E. Chadeyras A. Aba-Adulugba E. Libeau G.. (2009). Capripoxvirus G-protein-coupled chemokine receptor: a host-range gene suitable for virus animal origin discrimination. J. Gen. Virol. 90, 1967–1977. doi: 10.1099/vir.0.010686-0, PMID: 19339476

[ref38] Lemoine F. Correia D. Lefort V. Doppelt-Azeroual O. Mareuil F. Cohen-Boulakia S.. (2019). NGPhylogeny.fr: new generation phylogenetic services for non-specialists. Nucleic Acids Res. 47, W260–W265. doi: 10.1093/nar/gkz303, PMID: 31028399 PMC6602494

[ref39] Letunic I. Bork P. (2021). Interactive tree of life (iTOL) v5: an online tool for phylogenetic tree display and annotation. Nucleic Acids Res. 49, W293–W296. doi: 10.1093/nar/gkab301, PMID: 33885785 PMC8265157

[ref40] Li B. Wu H. Miao Z. Hu L. Zhou L. Lu Y. (2022). Codon usage of hepatitis E viruses: a comprehensive analysis. Front. Microbiol. 13:938651. doi: 10.3389/fmicb.2022.938651, PMID: 35801104 PMC9253588

[ref41] Lu G. Xie J. Luo J. Shao R. Jia K. Li S. (2021). Lumpy skin disease outbreaks in China, since 3 August 2019. Transbound. Emerg. Dis. 68, 216–219. doi: 10.1111/tbed.13898, PMID: 33119963

[ref42] Ma J. Yuan Y. Shao J. Sun M. He W. Chen J.. (2022). Genomic characterization of lumpy skin disease virus in southern China. Transbound. Emerg. Dis. 69, 2788–2799. doi: 10.1111/tbed.14432, PMID: 34927369

[ref43] Mareuil F. Doppelt-Azeroual O. Ménager H. (2017). A public Galaxy platform at Pasteur used as an execution engine for web services. F1000Research. doi: 10.7490/f1000research.1114334.1

[ref44] Martin D. P. Murrell B. Golden M. Khoosal A. Muhire B. (2015). RDP4: detection and analysis of recombination patterns in virus genomes. Virus Evol. 1. doi: 10.1093/ve/vev003, PMID: 27774277 PMC5014473

[ref45] Mathijs E. Vandenbussche F. Nguyen L. Aerts L. Nguyen T. de Leeuw I.. (2021). Coding-complete sequences of recombinant lumpy skin disease viruses collected in 2020 from four outbreaks in northern Vietnam. Microbiol. Resour. Announc. 10:e0089721. doi: 10.1128/MRA.00897-21, PMID: 34854705 PMC8638603

[ref46] Mauldin M. R. McCollum A. M. Nakazawa Y. J. Mandra A. Whitehouse E. R. Davidson W.. (2022). Exportation of Monkeypox virus from the African continent. J. Infect. Dis. 225, 1367–1376. doi: 10.1093/infdis/jiaa559, PMID: 32880628 PMC9016419

[ref47] Morgenstern M. Klement E. (2020). The effect of vaccination with live attenuated Neethling lumpy skin disease vaccine on milk production and mortality-an analysis of 77 dairy farms in Israel. Vaccines 8. doi: 10.3390/vaccines8020324, PMID: 32575395 PMC7350216

[ref48] Namazi F. Khodakaram Tafti A. (2021). Lumpy skin disease, an emerging transboundary viral disease: a review. Vet. Med. Sci. 7, 888–896. doi: 10.1002/vms3.434, PMID: 33522708 PMC8136940

[ref49] Ota T. Nei M. (1994). Variance and covariances of the numbers of synonymous and nonsynonymous substitutions per site. Mol. Biol. Evol. 11, 613–619. doi: 10.1093/oxfordjournals.molbev.a040140, PMID: 8078400

[ref50] Pecori R. Di Giorgio S. Paulo Lorenzo J. Nina Papavasiliou F. (2022). Functions and consequences of AID/APOBEC-mediated DNA and RNA deamination. Nat. Rev. Genet. 23, 505–518. doi: 10.1038/s41576-022-00459-8 35256818 PMC8900473

[ref51] Rose P. P. Korber B. T. (2000). Detecting hypermutations in viral sequences with an emphasis on G → A hypermutation. Bioinformatics 16, 400–401. doi: 10.1093/bioinformatics/16.4.400 10869039

[ref52] Rozas J. Ferrer-Mata A. Sánchez-DelBarrio J. C. Guirao-Rico S. Librado P. Ramos-Onsins S. E.. (2017). DnaSP 6: DNA sequence polymorphism analysis of large data sets. Mol. Biol. Evol. 34, 3299–3302. doi: 10.1093/molbev/msx248, PMID: 29029172

[ref53] Senkevich T. G. Yutin N. Wolf Y. I. Koonin E. V. Moss B. (2021). Ancient gene capture and recent gene loss shape the evolution of orthopoxvirus-host interaction genes. mBio 12:e0149521. doi: 10.1128/mBio.01495-21 34253028 PMC8406176

[ref54] Senkevich T. G. Zhivkoplias E. K. Weisberg A. S. Moss B. (2020). Inactivation of genes by frameshift mutations provides rapid adaptation of an attenuated vaccinia virus. J. Virol. 94. doi: 10.1128/JVI.01053-20, PMID: 32669330 PMC7459559

[ref55] Sevik M. Dogan M. (2017). Epidemiological and molecular studies on lumpy skin disease outbreaks in Turkey during 2014–2015. Transbound. Emerg. Dis. 64, 1268–1279. doi: 10.1111/tbed.12501, PMID: 27039847

[ref56] Sheikh A. Al-Taher A. Al-Nazawi M. Al-Mubarak A. I. Kandeel M. (2020). Analysis of preferred codon usage in the coronavirus N genes and their implications for genome evolution and vaccine design. J. Virol. Methods 277:113806. doi: 10.1016/j.jviromet.2019.113806, PMID: 31911390 PMC7119019

[ref57] Shumilova I. Nesterov A. Byadovskaya O. Prutnikov P. Wallace D. B. Mokeeva M.. (2022). A recombinant vaccine-like strain of lumpy skin disease virus causes low-level infection of cattle through virus-inoculated feed. Pathogens 11. doi: 10.3390/pathogens11080920, PMID: 36015041 PMC9414542

[ref58] Sprygin A. Babin Y. Pestova Y. Kononova S. Wallace D. B. van Schalkwyk A.. (2018a). Analysis and insights into recombination signals in lumpy skin disease virus recovered in the field. PLoS One 13:e0207480. doi: 10.1371/journal.pone.0207480, PMID: 30540759 PMC6291113

[ref59] Sprygin A. Pestova Y. Bjadovskaya O. Prutnikov P. Zinyakov N. Kononova S.. (2020a). Evidence of recombination of vaccine strains of lumpy skin disease virus with field strains, causing disease. PLoS One 15:e0232584. doi: 10.1371/journal.pone.0232584, PMID: 32401805 PMC7219772

[ref60] Sprygin A. Pestova Y. Prutnikov P. Kononov A. (2018b). Detection of vaccine-like lumpy skin disease virus in cattle and *Musca domestica* L. flies in an outbreak of lumpy skin disease in Russia in 2017. Transbound. Emerg. Dis. 65, 1137–1144. doi: 10.1111/tbed.12897 29932512

[ref61] Sprygin A. van Schalkwyk A. Shumilova I. Nesterov A. Kononova S. Prutnikov P.. (2020b). Full-length genome characterization of a novel recombinant vaccine-like lumpy skin disease virus strain detected during the climatic winter in Russia, 2019. Arch. Virol. 165, 2675–2677. doi: 10.1007/s00705-020-04756-7, PMID: 32772251

[ref62] Tasioudi K. E. Antoniou S. E. Iliadou P. Sachpatzidis A. Plevraki E. Agianniotaki E. I.. (2016). Emergence of lumpy skin disease in Greece, 2015. Transbound. Emerg. Dis. 63, 260–265. doi: 10.1111/tbed.12497, PMID: 26991342

[ref63] Tian H. F. Hu Q. M. Xiao H. B. Zeng L. B. Meng Y. Li Z. (2020). Genetic and codon usage bias analyses of major capsid protein gene in Ranavirus. Infect. Genet. Evol. 84:104379. doi: 10.1016/j.meegid.2020.104379, PMID: 32497680

[ref64] Tran H. T. T. Truong A. D. Dang A. K. Ly D. V. Nguyen C. T. Chu N. T.. (2021). Lumpy skin disease outbreaks in vietnam, 2020. Transbound. Emerg. Dis. 68, 977–980. doi: 10.1111/tbed.14022, PMID: 33548101

[ref65] Tuppurainen E. Dietze K. Wolff J. Bergmann H. Beltran-Alcrudo D. Fahrion A.. (2021). Review: vaccines and vaccination against lumpy skin disease. Vaccines 9. doi: 10.3390/vaccines9101136, PMID: 34696244 PMC8539040

[ref66] Tuppurainen E. S. Pearson C. R. Bachanek-Bankowska K. Knowles N. J. Amareen S. Frost L.. (2014). Characterization of sheep pox virus vaccine for cattle against lumpy skin disease virus. Antivir. Res. 109, 1–6. doi: 10.1016/j.antiviral.2014.06.009, PMID: 24973760 PMC4149609

[ref67] van Schalkwyk A. Byadovskaya O. Shumilova I. Wallace D. B. Sprygin A. (2022). Estimating evolutionary changes between highly passaged and original parental lumpy skin disease virus strains. Transbound. Emerg. Dis. 69, e486–e496. doi: 10.1111/tbed.14326, PMID: 34555250

[ref68] van Schalkwyk A. Kara P. Ebersohn K. Mather A. Annandale C. H. Venter E. H.. (2020). Potential link of single nucleotide polymorphisms to virulence of vaccine-associated field strains of lumpy skin disease virus in South Africa. Transbound. Emerg. Dis. 67, 2946–2960. doi: 10.1111/tbed.13670, PMID: 32506755 PMC9292827

[ref69] Vandenbussche F. Mathijs E. Haegeman A. Al-Majali A. van Borm S. de Clercq K. (2016). Complete genome sequence of capripoxvirus strain KSGP 0240 from a commercial live attenuated vaccine. Genome Announc. 4. doi: 10.1128/genomeA.01114-16, PMID: 27795268 PMC5073255

[ref70] Vandenbussche F. Mathijs E. Philips W. Saduakassova M. de Leeuw I. Sultanov A.. (2022). Recombinant LSDV strains in Asia: vaccine spillover or natural emergence? Viruses 14. doi: 10.3390/v14071429, PMID: 35891412 PMC9318037

[ref71] Vetrivel U. Arunkumar V. Dorairaj S. (2007). ACUA: a software tool for automated codon usage analysis. Bioinformation 2, 62–63. doi: 10.6026/97320630002062, PMID: 18188422 PMC2174420

[ref72] Vilsker M. Moosa Y. Nooij S. Fonseca V. Ghysens Y. Dumon K.. (2019). Genome detective: an automated system for virus identification from high-throughput sequencing data. Bioinformatics 35, 871–873. doi: 10.1093/bioinformatics/bty695, PMID: 30124794 PMC6524403

[ref73] Wallace D. B. Viljoen G. J. (2005). Immune responses to recombinants of the South African vaccine strain of lumpy skin disease virus generated by using thymidine kinase gene insertion. Vaccine 23, 3061–3067. doi: 10.1016/j.vaccine.2004.10.006, PMID: 15811653

[ref74] Wang L. Xing H. Yuan Y. Wang X. Saeed M. Tao J.. (2018). Genome-wide analysis of codon usage bias in four sequenced cotton species. PLoS One 13:e0194372. doi: 10.1371/journal.pone.0194372 29584741 PMC5870960

[ref75] Wang J. Xu Z. Wang Z. Li Q. Liang X. Ye S.. (2022). Isolation, identification and phylogenetic analysis of lumpy skin disease virus strain of outbreak in Guangdong, China. Transbound. Emerg. Dis. 69, e2291–e2301. doi: 10.1111/tbed.14570 35478381

[ref76] Warren C. J. Xu T. Guo K. Griffin L. M. Westrich J. A. Lee D.. (2015). APOBEC3A functions as a restriction factor of human papillomavirus. J. Virol. 89, 688–702. doi: 10.1128/JVI.02383-14, PMID: 25355878 PMC4301161

[ref77] Yinka-Ogunleye A. Aruna O. Dalhat M. Ogoina D. McCollum A. Disu Y.. (2019). Outbreak of human monkeypox in Nigeria in 2017–18: a clinical and epidemiological report. Lancet Infect. Dis. 19, 872–879. doi: 10.1016/S1473-3099(19)30294-4, PMID: 31285143 PMC9628943

[ref78] Young E. Basson P. A. Weiss K. E. (1970). Experimental infection of game animals with lumpy skin disease virus (prototype strain Neethling). Onderstepoort J. Vet. Res. 37, 79–87. 5535829

[ref79] Zhao Y. Zheng H. Xu A. Yan D. Jiang Z. Qi Q.. (2016). Analysis of codon usage bias of envelope glycoprotein genes in nuclear polyhedrosis virus (NPV) and its relation to evolution. BMC Genomics 17:677. doi: 10.1186/s12864-016-3021-7, PMID: 27558469 PMC4997668

