# Supplementary figures and images for: Sign of APOBEC editing, purifying selection, frameshift, and in-frame nonsense mutations in the microevolution of lumpy skin disease virus
Source: Front Microbiol. 2023 Nov 14;14:1214414. doi: 10.3389/fmicb.2023.1214414 (PMC10682384; doi:10.3389/fmicb.2023.1214414)

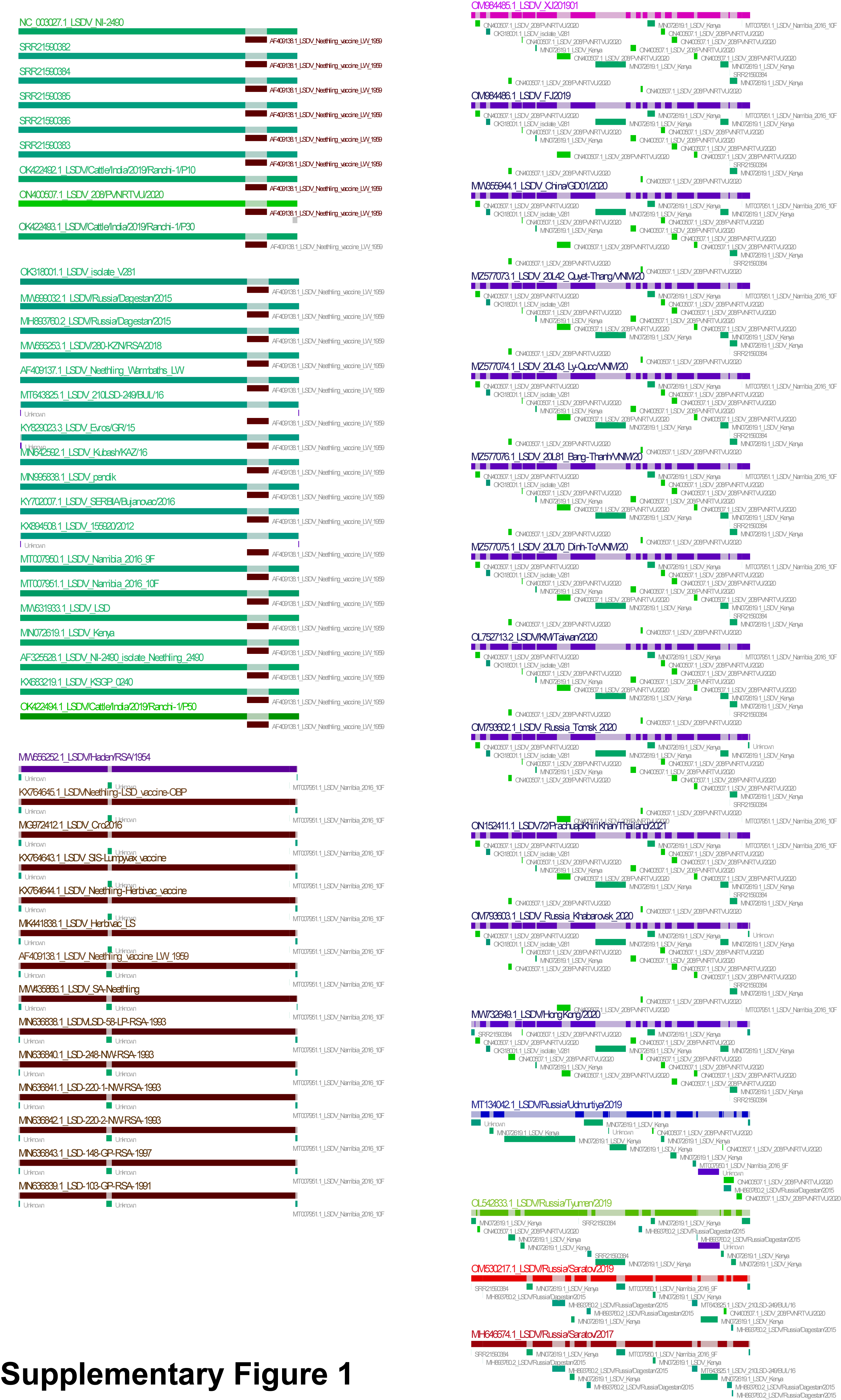

Supplement: Supplementary Figure S1 — A recombination detection program (RDP) analysis based on potential recombination events is depicted for each virus. [file Image_1.TIF]

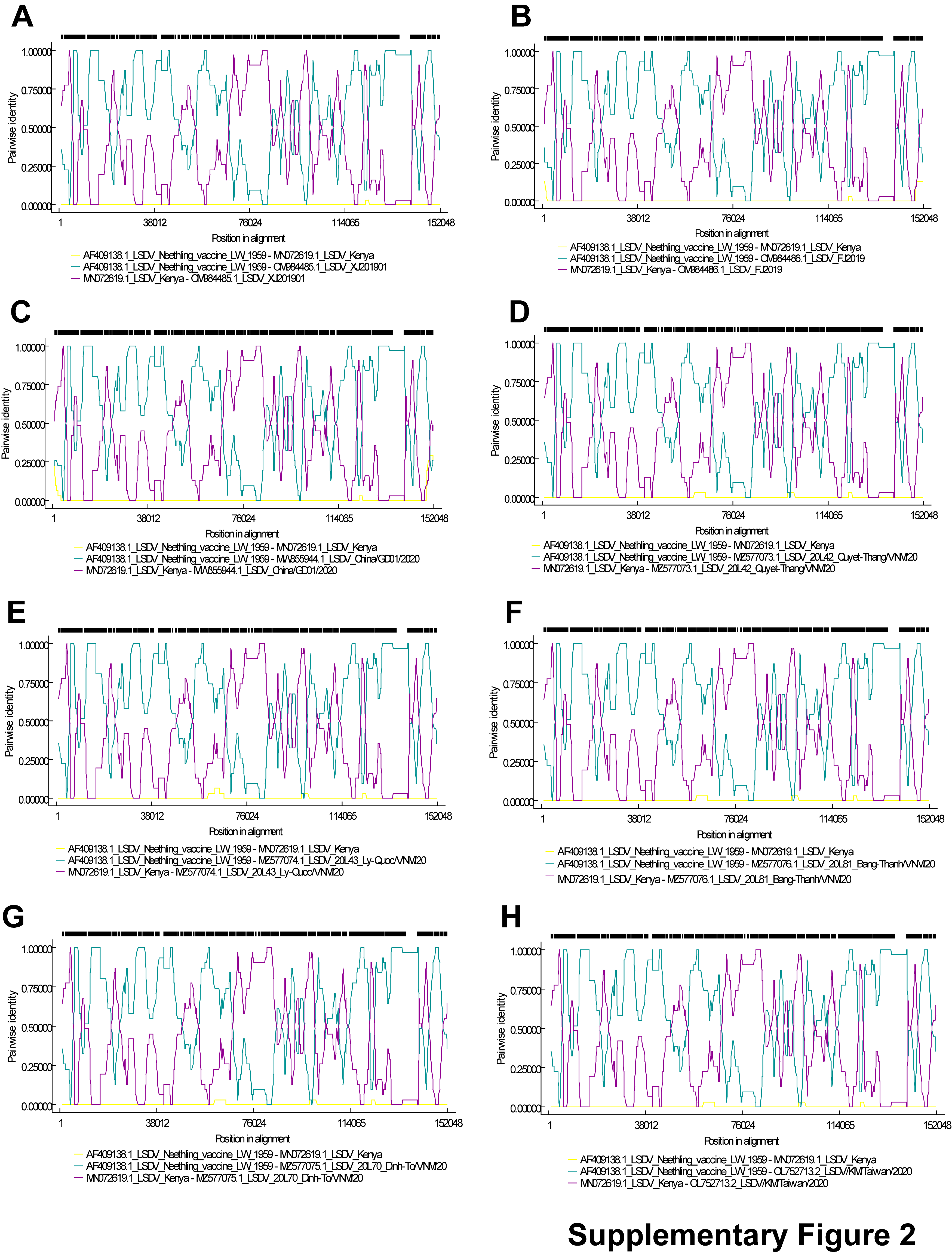

Supplement: Supplementary Figure S2 — Graphical representation of potential recombination events in (A) OM984485.1/LSDV/XJ201901; (B) OM984486.1/LSDV/FJ2019; (C) MW355944.1/LSDV/ China/GD01/2020; (D) MZ577073.1/LSDV/20L42/Quyet-Thang/VNM/20; (E) MZ577074.1/LSDV_20L43/Ly-Quoc/VNM/20; (F) MZ577076.1_LSDV_20L81_Bang-Thanh/VNM/20; (G) MZ577075.1_LSDV_20L70_Dinh-To/VNM/20; and (H) OL752713.2/LSDV/KM/Taiwan/2020. [file Image_2.TIF]

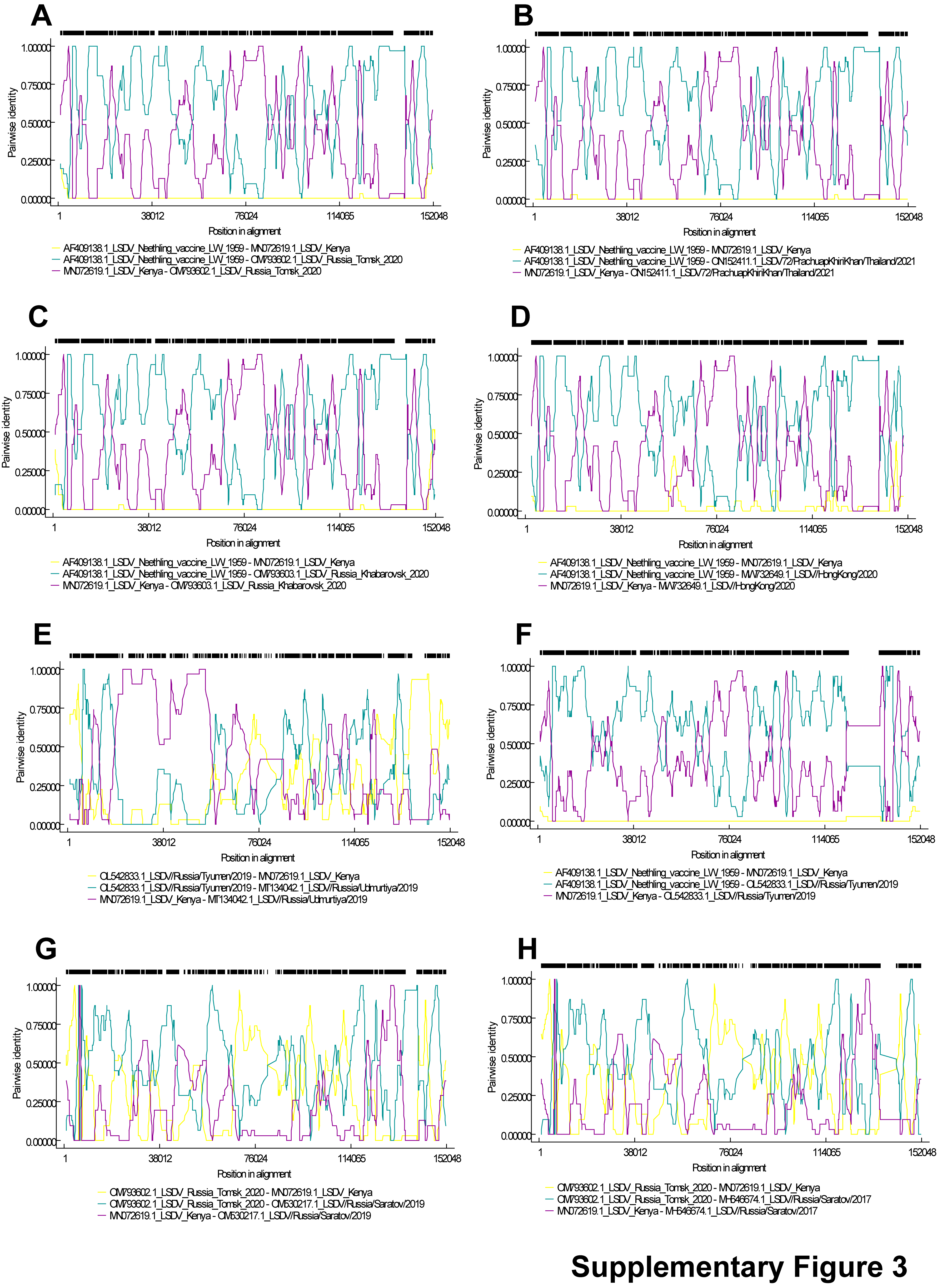

Supplement: Supplementary Figure S3 — Graphical representation of potential recombination events in (A) OM793602.1_LSDV_Russia_Tomsk_2020; (B) ON152411.1_LSDV72/PrachuapKhiriKhan/Thailand/2021; (C) OM793603.1_LSDV_Russia_Khabarovsk_2020; (D) MW732649.1/ LSDV/HongKong/2020; (E) MT134042.1_LSDV/Russia/Udmurtiya/2019; (F) OL542833.1/ LSDV/Russia/Tyumen/2019; (G) OM530217.1_LSDV/Russia/Saratov/2019; and (H) MH646674.1/LSDV/Russia/Saratov/2017. [file Image_3.TIF]

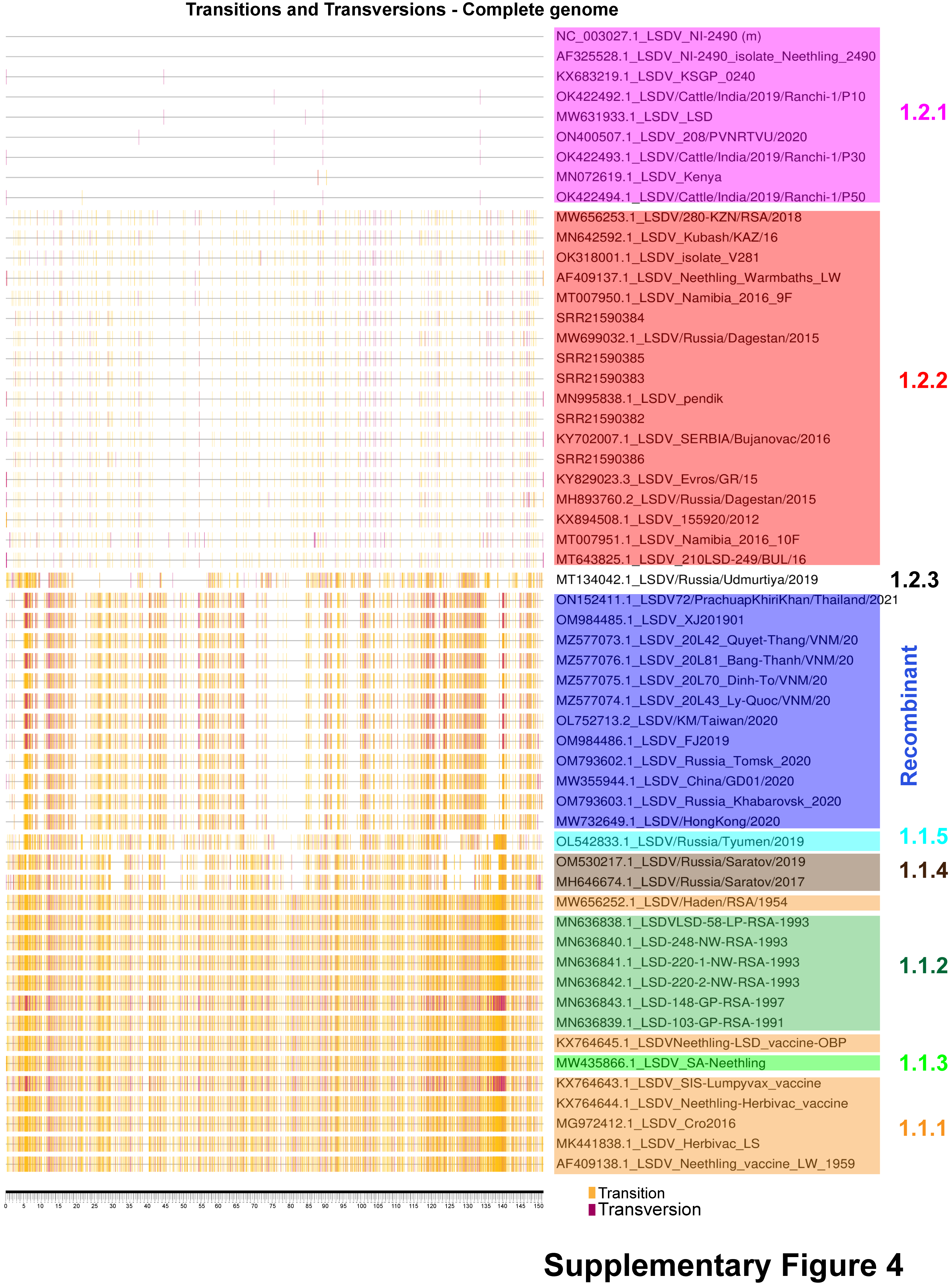

Supplement: Supplementary Figure S4 — Transitions and Transversion mutations at the complete genome levels of different clusters of LSD viruses were visualized, and the NC_003027.1 sequence was used as a reference in this analysis. [file Image_4.TIF]

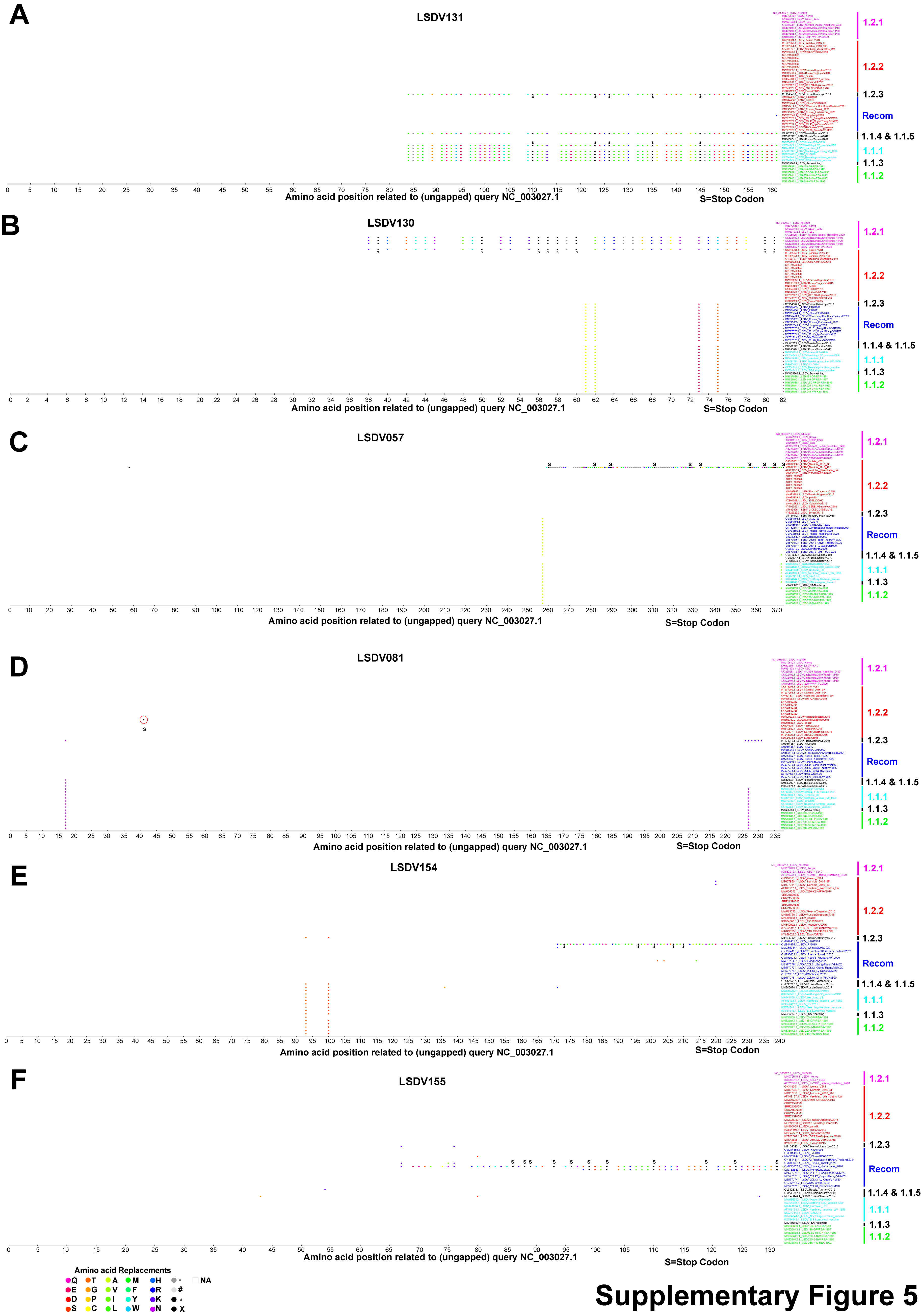

Supplement: Supplementary Figure S5 — In-frame nonsense mutations in LSDV131, LSDV130, LSDV057, LSDV081, LSDV154 and LSDV155 genes. In-frame nonsense mutations in the gene (A) LSDV131; (B) LSDV130; (C) LSDV057; (D) LSDV081; (E) LSDV154 and (F) LSDV155. Details of sequences used in this analysis are provided in Supplementary Data 1. [file Image_5.TIF]

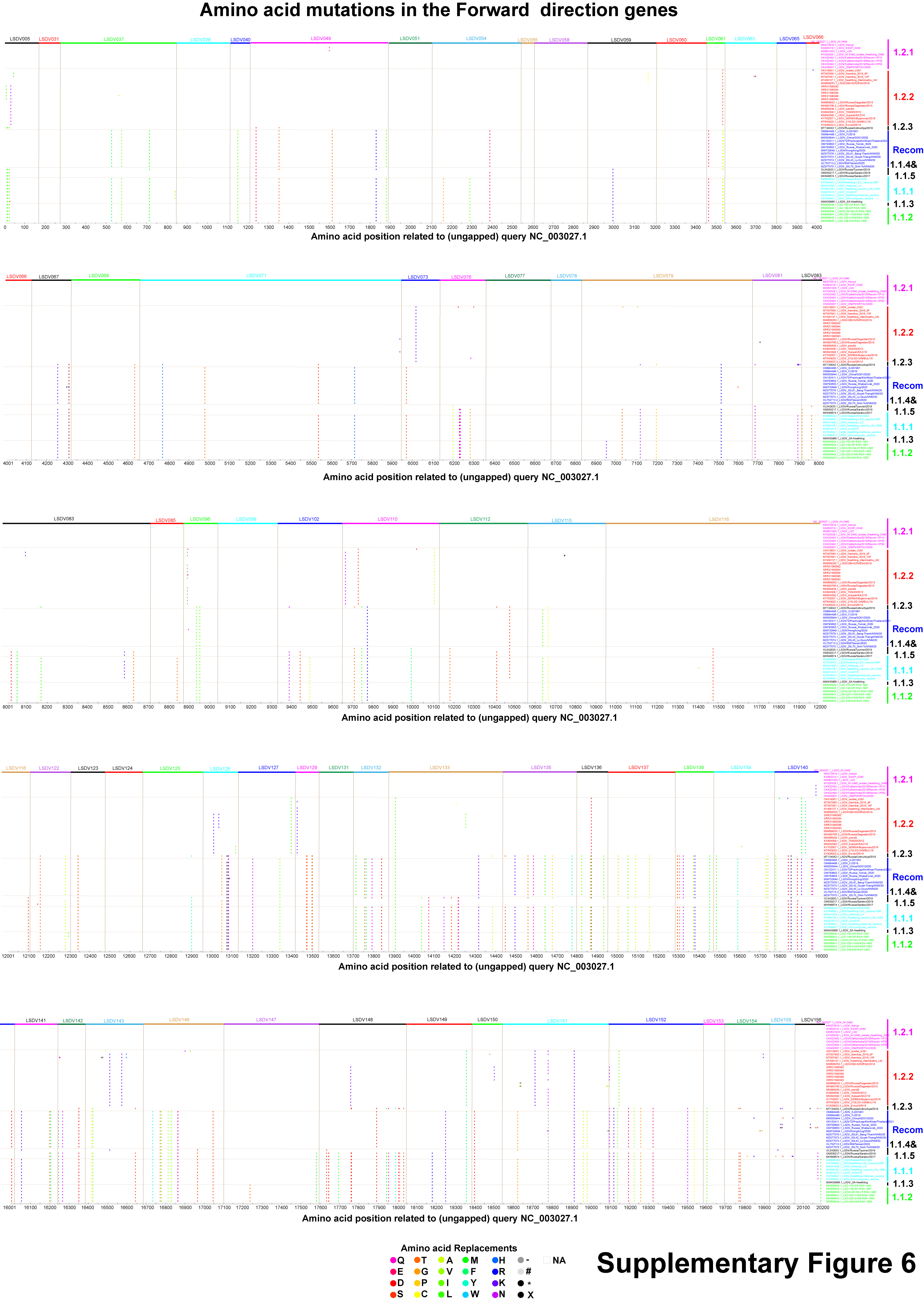

Supplement: Supplementary Figure S6 — The amino acid mutations in the forward direction transcribe genes coding regions from the center to ITR regions of different clusters of LSD viruses. The NC_003027.1 sequence was used as a reference in this analysis. [file Image_6.TIF]

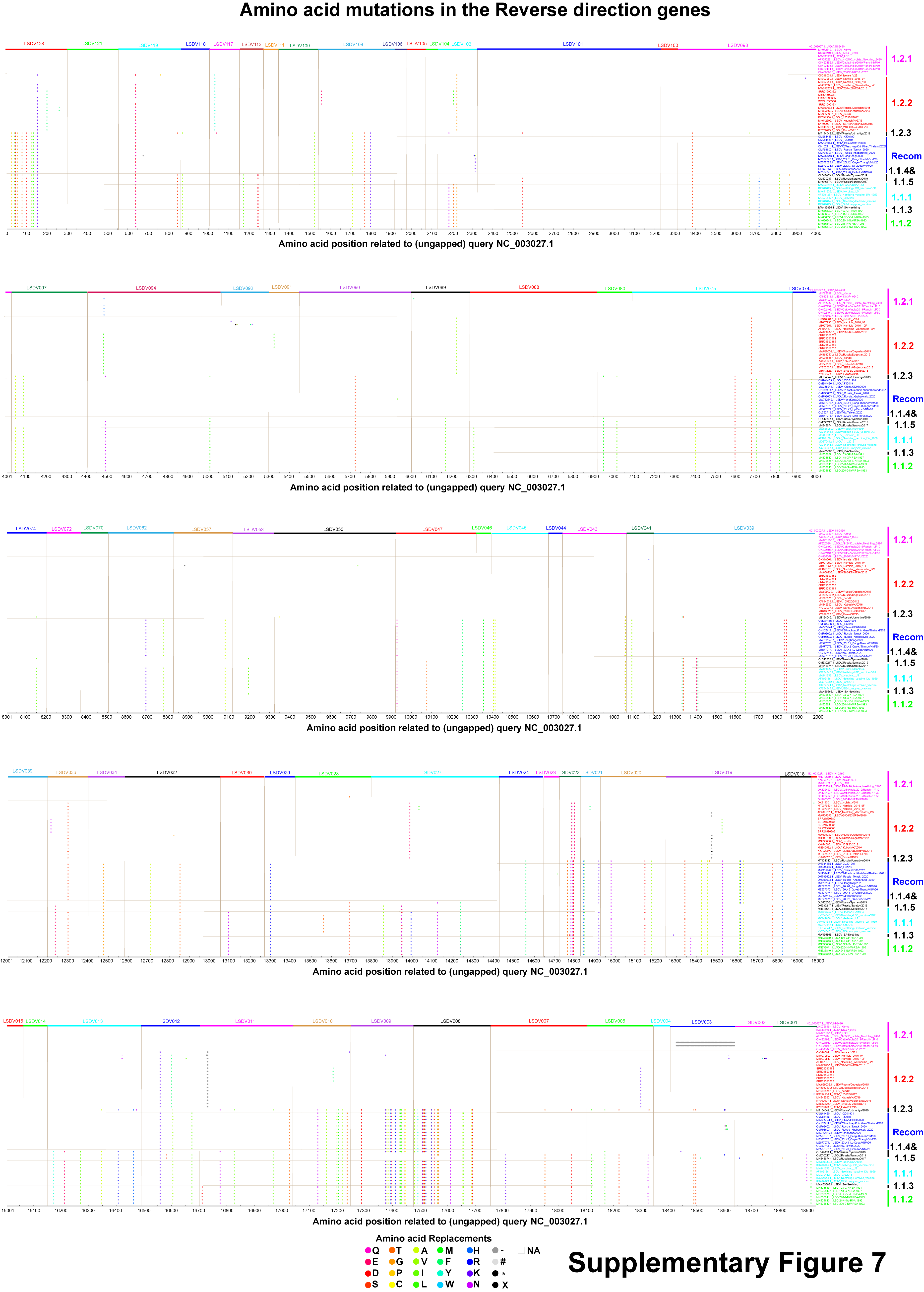

Supplement: Supplementary Figure S7 — The amino acid mutations in the reverse direction transcribe genes coding regions from the center to ITR regions of different clusters of LSD viruses. The NC_003027.1 sequence was used as a reference in this analysis. [file Image_7.TIF]

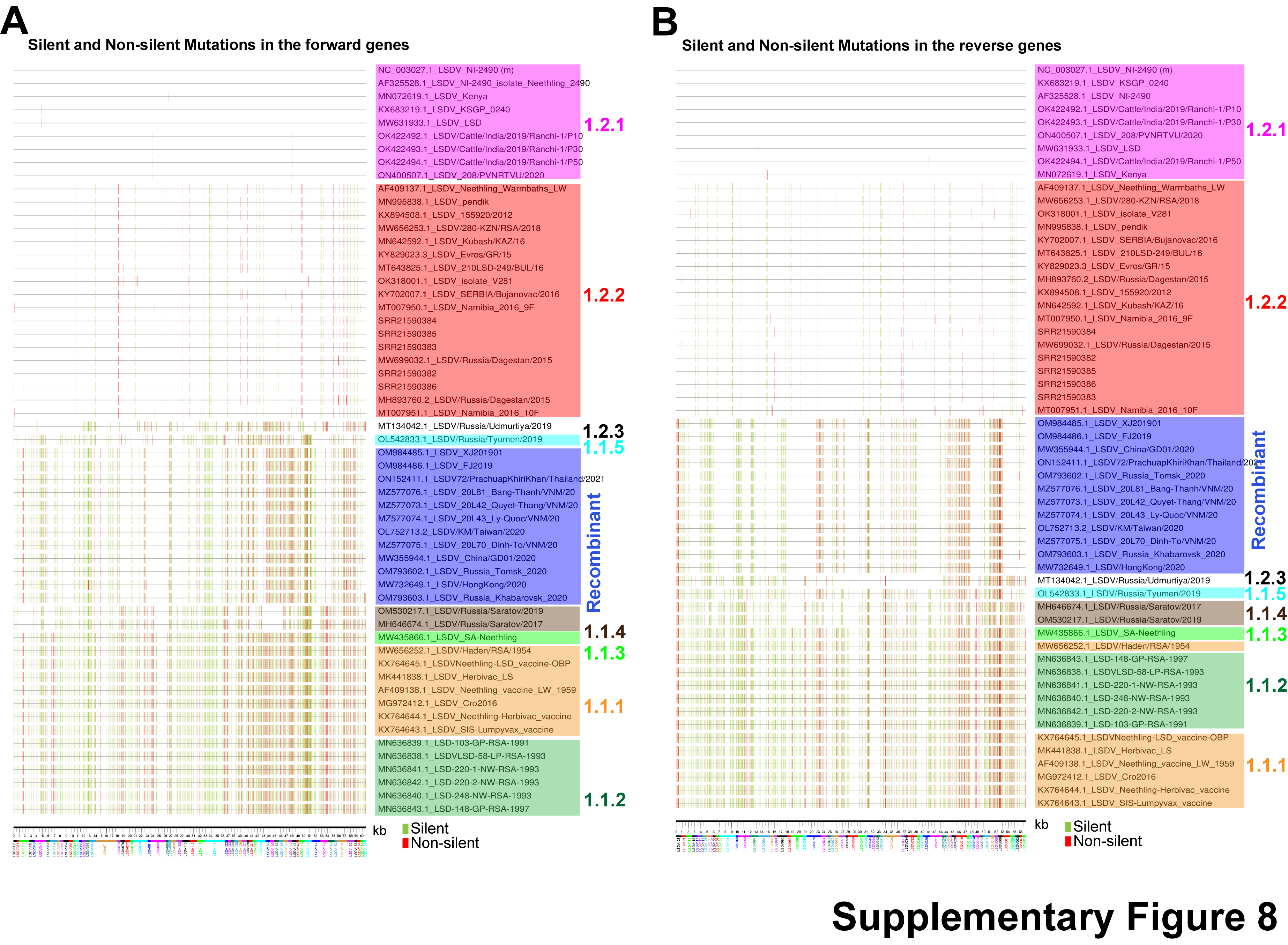

Supplement: Supplementary Figure S8 — (A,B) Silent and Non-silent mutations in the coding regions of LSD viruses were visualized, (A) genes that are transcribing in the forward direction and (B) genes that are transcribing in the reverse direction. The NC_003027.1 sequence was used as a reference in this analysis. [file Image_8.TIF]

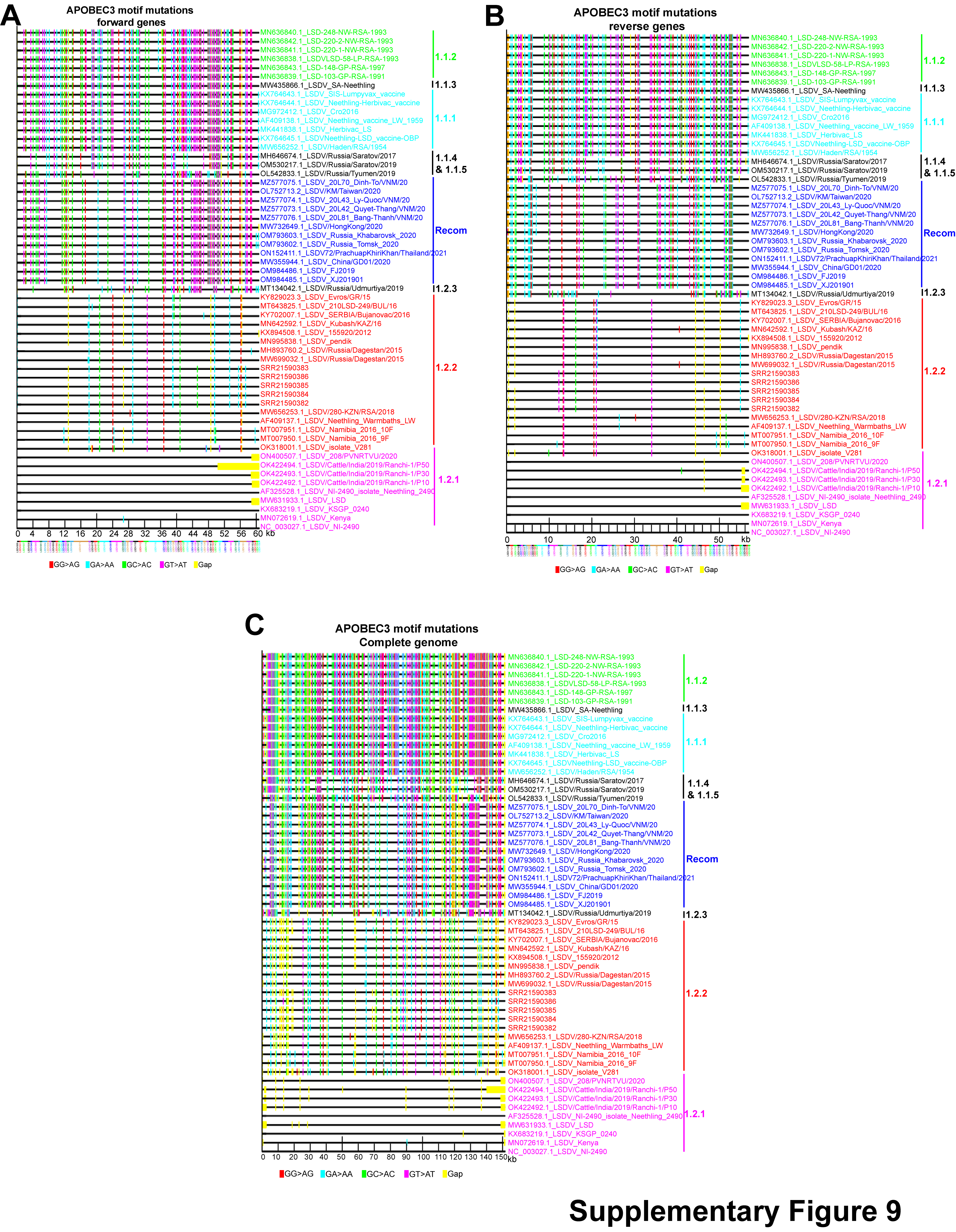

Supplement: Supplementary Figure S9 — The APOBEC motif mutations in the different clusters of LSD viruses were visualized, (A) genes that are transcribing in the forward direction, (B) genes that are transcribing in the reverse direction, and (C) at the complete genome levels. The NC_003027.1 sequence was used as a reference in this analysis. [file Image_9.TIF]

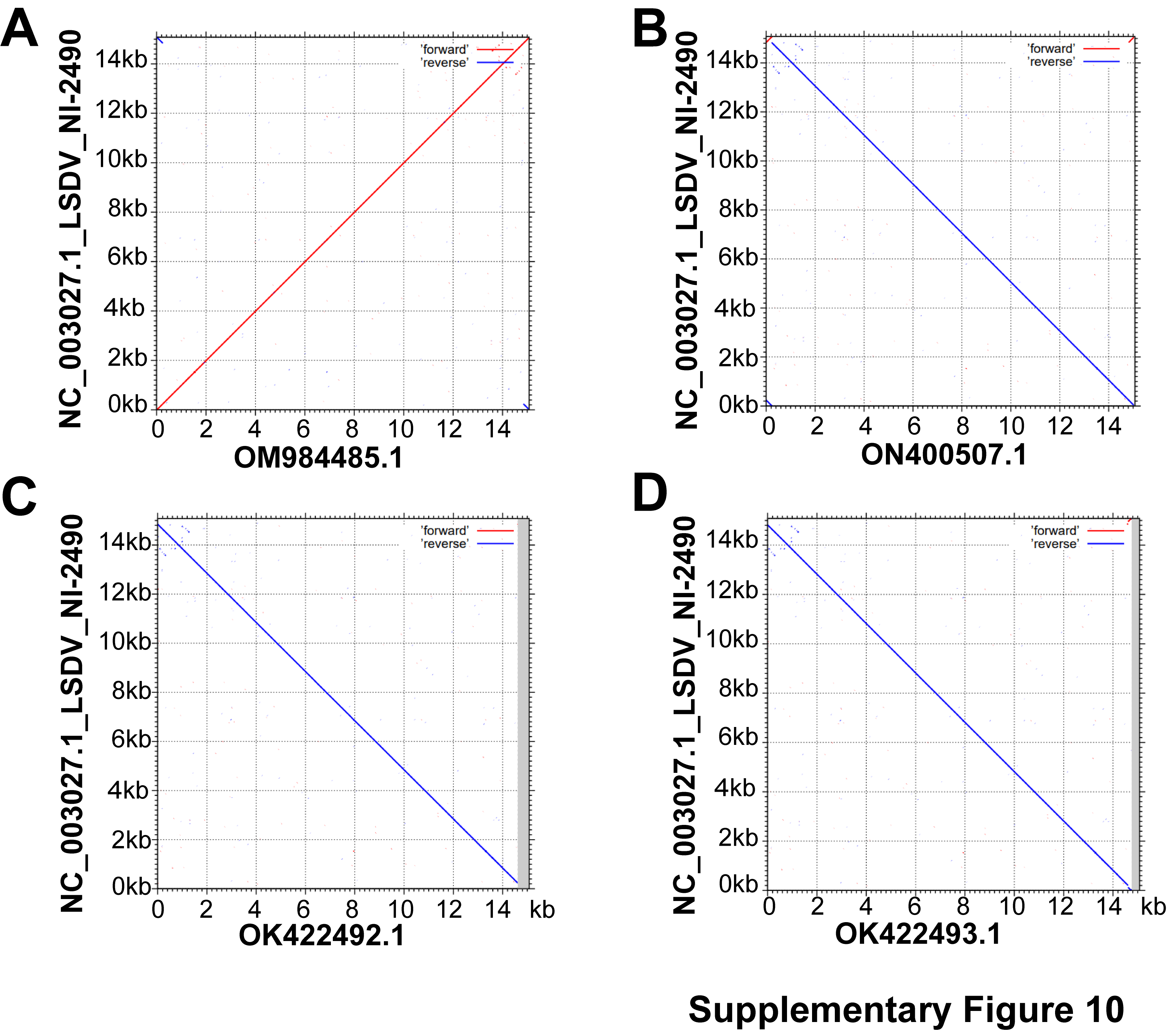

Supplement: Supplementary Figure S10 — (A–D) The Plot LAST hits analysis shows the OK422492.1/India/2019/Ranchi-1/P10, OK422493.1//India/2019/Ranchi-1/P3, and ON400507.1/208/PVNRTVU/202 sequences were submitted as reverse complement compared to NCBI reference sequences NC_003027.1_LSDV_NI-249. [file Image_10.TIF]
